# The Laryngovibrogram as a normalized spatiotemporal representation of vocal fold dynamics

**DOI:** 10.1038/s41598-025-00966-8

**Published:** 2025-05-12

**Authors:** Mona Kirstin Fehling, Maria Schuster, Maximilian Linxweiler, Jörg Lohscheller

**Affiliations:** 1 Department of Computer Science, Trier University of Applied Sciences, Schneidershof, 54293 Trier, Germany; 2 Department of Otorhinolaryngology, Head and Neck Surgery, Saarland University Medical Center / Saarland University Faculty of Medicine, 66421 Homburg/Saar, Germany; 3 Department of Otorhinolaryngology and Head and Neck Surgery, Ludwig Maximilian University of Munich, 81377 Munich, Germany

**Keywords:** Laryngovibrogram, Phonovibrogram, Laryngeal high-speed videoendoscopy, Voice disorders, Diseases, Health care, Medical research

## Abstract

Laryngeal high-speed video (HSV)-endoscopy allows for fast, non-invasive diagnosis of voice disorders and forms the basis for a comprehensive quantitative analysis of the vocal folds’ (VFs’) spatiotemporal vibrational behavior. Previous approaches, such as the Phonovibrogram (PVG), describe the vibrational behavior of vocal folds (VFs) based exclusively on the time-varying glottal opening. However, focusing solely on the glottal area overlooks the full extent and dynamic behavior of the VF tissue, factors that are crucial for the voice production process. This complicates clinical interpretation and, thus, the comparability of vibrational dynamics in both cross-sectional and longitudinal interventional studies. To address these limitations, this work aims to extend the PVG to provide a more comprehensive representation of the vibrational behavior across the entire VF tissue. Here, we present the Laryngovibrogram (LVG), which is obtained by segmenting not only the glottal area but also the VFs’ tissue, providing a compact quantitative representation of the VFs’ vibrational behavior. The potential of the proposed LVG representation was investigated on 73 HSV recordings from healthy (38 HSVs) and pathological subjects (35 HSVs) in stationary as well as non-stationary phonations. It is demonstrated that the LVG reliably maps the vibrational behavior along the entire length of the VFs tissue for both physiological and pathological phonations. Compared to PVG-based measures, LVG-based measures exhibited greater stability in healthy subjects, allowing for a narrower normative range, and showed stronger effect sizes in differentiating clinical groups, suggesting a more robust assessment of vibratory impairments. By scaling the vibration amplitude relative to the length of the segmented VF tissue, the VF vibrations are normalized, enabling meaningful quantitative intra- and inter-individual comparisons. Additionally, calculating the angle enclosed by the two VFs makes it possible to analyze transient effects that occur during non-stationary phonation maneuvers, such as voice onset. By integrating information about the VF tissue, the LVG introduced here represents a paradigm shift in the analysis of laryngeal dynamics from focusing solely on the glottal area to a holistic analysis of the entire VF kinematics, which might improve pathology detection accuracy, reduce subjective assessment errors, and optimize treatment follow-ups, ultimately enhancing both clinical diagnostics and therapeutic outcomes.

## Introduction

Dysphonia is a frequent disorder, affecting approximately 30% of the adult population in the United States at some point during their lifetime, with 7.6% of individuals experiencing voice problems at any given time^1,2^. Since voice is the foundation of human verbal communication, voice disorders have far-reaching implications on the individuals’ personal, social, and professional lives. This is becoming increasingly important as an ever-growing number of today’s jobs rely on well-functioning communication skills. Moreover, work-related absences due to voice disorders, along with the need for medical consultation, result in high socio-economic costs each year^1^. Therefore, early diagnosis and comprehensive assessment of voice therapy outcomes are crucial for both the individual and society.

The human voice is generated in the larynx by the exhaled airstream from the lungs, which induces vibration in the pairwise-arranged vocal folds (VFs). While healthy voices are characterized by regular, symmetric, and synchronous VF vibrations, voice disorders arise from disturbances in the VFs’ vibrational behavior^3,4^. These disturbances can result from various medical conditions, including non-organic disorders like muscle tension dysphonia or VF paresis, as well as organic lesions such as VF polyps^3^. With typical fundamental frequencies in the range of approximately 80 to 250 Hz^3^, VF vibrations are too fast for direct visual assessment^5^. To overcome this challenge, various techniques have been developed to capture VF kinematics. A widespread technique is laryngeal high-speed videoendoscopy, which captures the VF vibrations in real-time at framerates of 2,000 to 20,000 fps^6–11^. The so captured high-speed video (HSV) data enable accurate quantification of VF kinematics, where the high temporal resolution of high-speed imaging allows for detecting of even slight variations in VF vibration^12–15^. Thus, HSVs lay the foundation for an objective analysis of the VFs’ vibrational behavior^4^. In addition to capturing this vibrational behavior, laryngeal imaging also allows for the observation of how the complex interplay among laryngeal muscles impacts the configuration of the larynx. This interaction influences both the tension and length of the VFs within the larynx, as well as their adduction and abduction, which affects the size and position of the glottal opening. Together, these factors directly affect voice production by modulating VF vibration, pitch and loudness. While inter-individual variations in laryngeal configuration and the vibrational behavior of the VFs, as seen in HSVs, result primarily from anatomical differences between individuals, intra-individual variations are mainly due to dynamic physiological changes such as vocal loading effects, as well as changes in laryngeal tension and the particular positioning of the endoscope within the orophaynx during imaging^3,16,17^. These intra-individual variations are thus considered normal to a certain degree^18–20^. Changes in the oropharyngeal endoscope position can also have a scaling effect, altering the visible size of anatomical structures in HSVs and affecting the observed vibrational amplitudes^17^. Without proper normalization, these variations complicate both intra- and inter-individual comparisons of VF dynamics. In previous works, VF deflections were deduced from the glottal opening and used as a basis for analyzing the VFs’ vibrational behavior^21–28^. This vibrational behavior of the VFs’, as captured in HSVs, has been shown to be effective in identifying underlying pathologies^29–37^. Consequently, various approaches for measuring laryngeal structures in HSV recordings have been proposed^38–42^. However, even comparing the vibrational behavior across different recordings from the same subject remains limited. This limitation in comparability complicates the evaluation of VFs’ vibrational behavior in clinical routine, praticularly during follow-up examinations, where VF vibrations from different recordings are compared. The application of laryngeal high-speed videoendoscopy in clinical routine is further impeded by the large amounts of data that are generated due to the high temporal resolution of the HSVs, which makes their evaluation a time-consuming task. Hence, several compact and clinically meaningful representations of VF dynamics have been developed^10,18,25,26,28,43–45^ to facilitate the rapid assessment of the VFs’ vibrational behavior from HSVs in clinical routine. One of the most comprehensive representations of VF dynamics is the Phonovibrogram (PVG), introduced by Lohscheller et al. in 2008^25^. Since the approach introduced in this work aims for extending the PVG, a brief outline of the PVG construction process is provided to facilitate understanding.

### Phonovibrogram

**Fig. 1:**
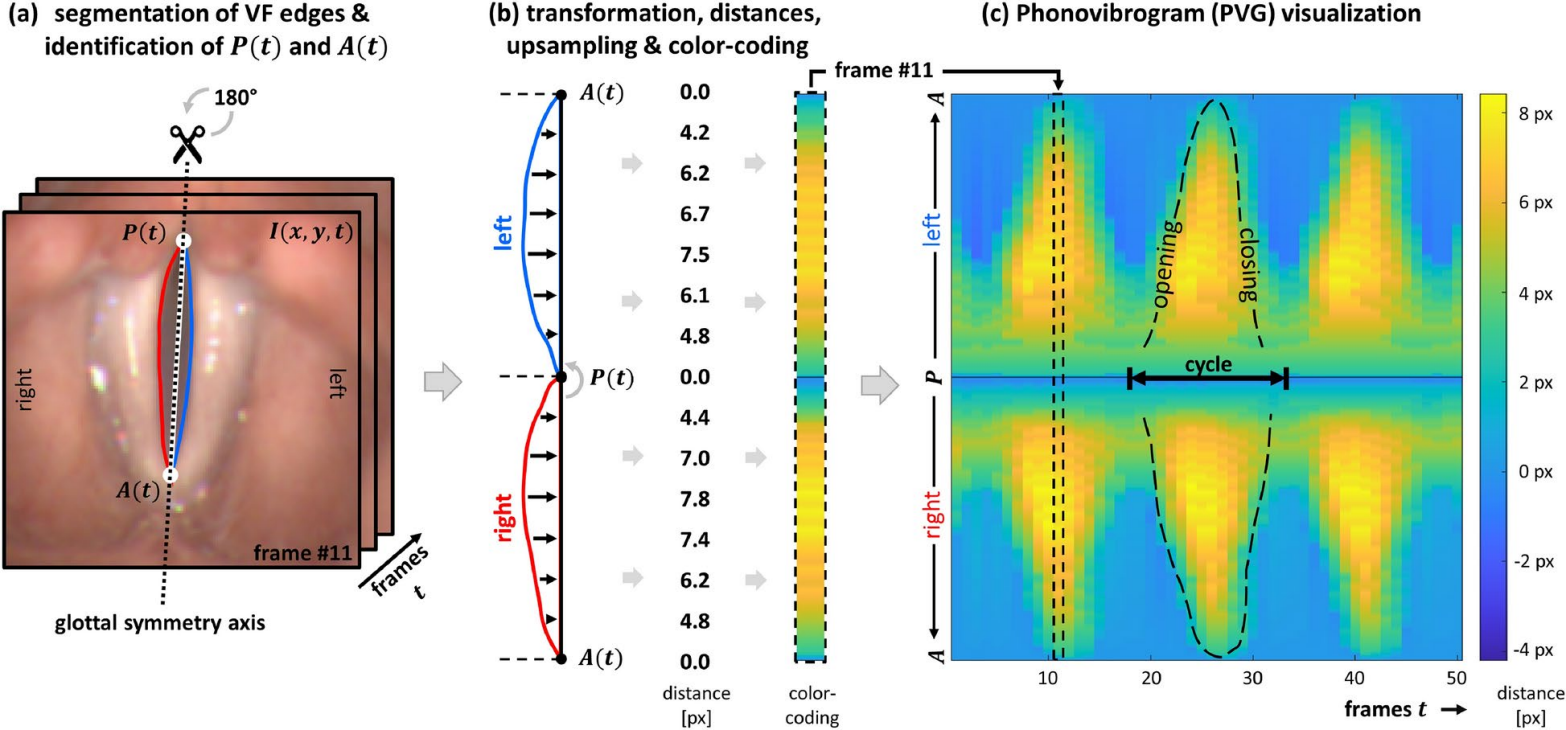
Computation of the PVG visualization depicting the VFs’ vibrational behavior from laryngeal HSVs. (**a**) Initial identification of the posterior point *P*(*t*) and anterior point *A*(*t*) from the segmented glottal area in each frame *I*(*x*, *y*, *t*). These points are used to define the glottal symmetry axis, based on which the outline of the segmented glottal area is split into left and right contours that represent the left and right edges of the VFs. (**b**) The left VF is then rotated 180$^{\circ }$ counter-clockwise around the point *P*(*t*). The distances between the contours and the glottal symmetry axis are computed and color-coded, resulting in one color strip per frame. (**c**) Concatenation of the color-coded distance strips for all frames of the sequence finally builds the PVG, where characteristic geometric shapes represent the VFs’ vibrational behavior.

The PVG provides a compact two-dimensional visualization of the VFs’ vibrational behavior in the range of the glottal opening by color-coding the time-varying deflections of both VFs individually^25,46^. Figure 1 illustrates the construction of the PVG. From the derived glottis contour, the points *P*(*t*) and *A*(*t*) are identified as approximations of the posterior and anterior commissures, respectively. These points define the position of the glottal symmetry axis, which serves as a reference axis in the construction of the PVG. Based on this glottal symmetry axis, the outline of the glottis is split into left and right contours, representing the left and right edges of the VFs tissue surrounding the glottis (Fig. 1(a)). Next, the left VF contour is rotated 180$^{\circ }$ counter-clockwise around the point *P*(*t*) and upsampled to 256 equidistant points. The distances between each VF contour and the glottal symmetry axis are then computed and color-coded, resulting in one color-coded distance strip per video frame (Fig. 1(b)). Concatenating these color strips along the time-axis builds the PVG. Depending on the individual VF vibrations, characteristic geometric shapes emerge in the PVG visualization, representing the VF dynamics in the range of the glottal opening (Fig. 1(c)). These characteristic geometric PVG shapes have been shown to align with the different vibration types defined within the *basic protocol for functional assessment of voice pathology*^47^, published by the European Laryngological Society (ELS)^25^. Furthermore, successful PVG-based classification of voice disorders^31,35^ highlights the potential for such a compact representation of VFs’ vibrational behavior to enhance objective clinical diagnosis. In addition to classification, PVG has also been shown to be clinically valuable for assessing surgical outcomes and tracking post-operative recovery^33^, analyzing voice dynamics under different loading conditions^16^, and evaluating a range of pathological voice types^48^.

**Fig. 2:**
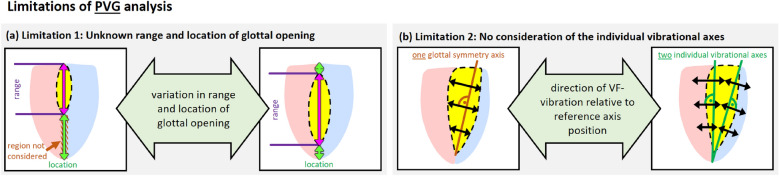
Limitations of PVG analysis that result from its restriction to the range of glottal opening and impede a comprehensive analysis of the VFs’ vibrational behavior. (**a**) Limitation 1: Unknown range and location of glottal opening. The exact range of the glottal opening remains unknown, which is particularly problematic in cases of incomplete glottal opening. Additionally, the location of the glottal opening relative to the full extent of the VFs remains unknown. (**b**) Limitation 2: No consideration of the individual vibrational axes. The glottal symmetry axis does not necessarily coincide with the VFs’ individual vibrational axes, which impacts the accuracy of the retrieved vibrational behavior. As a result, these limitations affect the interpretation of vibrational behavior with regard to comparability across different recordings and hinder the analysis of non-stationary phonation paradigms.

The PVG analysis, however, is systematically restricted due to the following limitations:

- **Limitation 1: Unknown range and location of glottal opening.** The PVG is derived from the contour of the segmented, time-varying glottal area. Therefore, the PVG is inextricably linked to the range of the glottal opening. However, for a comprehensive interpretation of the vibrational behavior of the VFs as a whole, understanding both the exact range and location of the glottal opening relative to the extent of the VF tissue is crucial. Restricting the analysis to the glottal opening is particularly problematic in cases of incomplete glottal opening, such as those caused by organic lesions, as it overlooks the actual extent of the VF tissue. Later sections examine a clinical example of a VF polyp, where incomplete glottal closure leads to a misleading PVG representation. (see Fig. 2(a))
- **Limitation 2: No consideration of the individual vibrational axes.** The glottal symmetry axis does not necessarily coincide with the VFs’ actual vibrational axes. This becomes particularly evident in pathologies such as unilateral VF paresis, where the affected VF may rest in the paramedian or intermediate position. Similarly, a VF polyp can alter the actual vibrational axis, especially if the lesion prevents complete glottic closure or induces localized vibration asymmetries. In such cases, analyzing the VFs’ vibrational behavior with respect to a single symmetry axis also impacts the representation of the non-affected side’s kinematics. Consequently, the morphoplogy of the motion trajectory may be altered if the VF deflections are not measured perpendicular to each VF’s specific vibrational axis. Later sections will illustrate this limitation with a clinical example of a VF polyp. (see Fig. 2(b))

These limitations also have far-reaching implications for interpreting the retrieved vibrational behavior, particularly regarding the comparability between different PVGs and the analysis of non-stationary phonation paradigms. Comparability between different PVGs is restricted because, without segmenting the VF tissue, the lengths of the VFs remain unknown. In such cases, quantitative intra- and inter-individual comparisons of HSV recordings based solely on the glottal opening are difficult, as crucial information about the VF tissue is missing. This difficulty in maintaining consistent conditions during assessments complicates the interpretation of follow-up examinations and further hinders the development of computer-aided systems for diagnosing voice disorders. Additionally, analyzing non-stationary phonation using the PVG representation is challenging, as non-stationary phonation cause considerable changes in the overall laryngeal configuration that affect the position and size of laryngeal structures in HSVs, which further complicates the accurate representation and analysis of the VFs vibrational behavior. To our knowledge, no prior studies have systematically investigated how these limitations impact clinical decision-making, primarily because conventional methods did not allow for the automatic extraction of the full VF tissue. By addressing this gap, our work introduces the LVG as a first step toward a more comprehensive representation of VF vibrations.

This work, therefore, aims to overcome the limitations of the PVG by extending it to offer a more comprehensive representation of the vibrational behavior across the entire VF tissue. Such an extension requires not only the segmentation of the glottis but also the segmentation of the entire VF tissue. The approach used for this purpose is briefly outlined below.

### Image-processing of laryngeal HSV

Robust extraction of the time-varying glottal area from laryngeal HSV frames is already challenging, even though the glottal area silhouettes clearly against the surrounding tissue and can, therefore, be extracted comparatively easily from the HSV recordings. Nevertheless, various approaches have been proposed to extract the glottal area. These approaches include thresholding techniques^13,49–53^, methods based on gray-level derivatives^54^, seeded region-growing procedures^55–57^, active contour models^58–60^, and segmentation using the watershed transform^61^. Typically, these methods require time-consuming manual user interactions, such as initial seeding or threshold selection. Moreover, human supervision and inspection of the achieved segmentation results are often necessary, as these methods are prone to errors. Despite extensive efforts, extracting the glottal area remains challenging. Moreover, none of the previously proposed methods extracts the VFs’ tissue itself.

However, with advancements made in Deep Learning over the past few years, Artificial Neural Networks (ANNs) have led to remarkable progress in various computer vision tasks. The application of Deep Learning has also extended into medical image processing, where ANNs have been applied to various tasks^62^. Some of the most popular tasks in medical image processing include enhancing the quality of medical data^63–67^, semantic segmentation of various anatomical structures^68–74^, cancer classification using different data modalities^75–80^, and, more recently, COVID-19 detection^81,82^.

First steps in utilizing ANNs for the segmentation of laryngeal structures were made by Rao et al., who successfully segmented the glottal area from laryngeal stroboscopic videos^83^, and Laves et al., who presented an approach for the segmentation of laryngeal structures and surgical tools from single images captured during surgery^84^. The application of the proposed approaches to laryngeal high-speed data is, however, limited. On the one hand, in contrast to single images, frames from HSV recordings usually exhibit reduced image quality due to lower spatial resolution and limitations in illumination. On the other hand, due to the HSVs’ high temporal resolution, special attention must be paid to the temporal aspect of VF motion to avoid discontinuities in segmentation results between consecutive frames. The first approaches to using an optimized U-Net for the segmentation of laryngeal HSVs were proposed by Fehling et al.^85^ and Kist et al.^86^. Kist et al. utilized an ANN for automatic glottis segmentation^86^, while Fehling et al. introduced a U-LSTM-based approach that, for the first time, extracts not only the glottal area from laryngeal HSV but also the oscillating VFs tissue itself^85^, thereby forming the basis for the work presented here. In this context, the term ’VF tissue’ refers specifically to the two-dimensional visible VF surface, as captured in HSV recordings. Thus, the extracted motion reflects only the VF dynamics of the visible portion within the endoscopic field of view, rather than their full three-dimensional motion. Consequently, the extracted VF contour does not contain information about potential superior-inferior shifts of the visible VF edge.

### This work

Here, we present a novel approach called ’Laryngovibrography’, which represents the dynamic information from laryngeal HSVs by considering not only the glottis but also the VF tissue itself. The resulting Laryngovibrogram (LVG) aims to provide a compact and normalized 2D representation of the VFs’ vibrational behavior, similar to the Phonovibrogram, but with the inclusion of the VF tissue itself. First, the procedure for constructing LVGs is presented, along with parameters for the quantitative description of the VFs’ vibrational dynamics retrieved from the LVGs. Following that, various applications of the LVG are evaluated, including assessing differences between the PVG and LVG representations of vibrational behavior, applying the LVG for both intra- and inter-individual comparisons of stationary HSV recordings, and exploring its potential for analyzing non-stationary phonations.

## Materials and methods

### Clinical data and data acquisition

The data used for method development and validation were obtained from the *Department of Otorhinolaryngology and Head and Neck Surgery* at the *University of Munich (Munich, Germany)* and the *Department of Otorhinolaryngology, Head and Neck Surgery* at the *Saarland University Medical Center (Homburg/Saar, Germany)*. The study was conducted in accordance with the Declaration of Helsinki, and ethical approval was obtained from the respective local ethics committees (*Ethikkommission bei der Medizinischen Fakultät der LMU München*, reference number: 391-14; and *Ethik-Kommission bei der Ärztekammer des Saarlandes*, reference number: 103/12), and all participants gave written informed consent prior to participation. Data analysis and visualizations were performed using *MATLAB* (*R2019a, The MathWorks Inc., Natick, MA, USA*)^87^.

Laryngeal HSVs were recorded in color using the rigid endoscopy system *HRES ENDOCAM 5562* from *Richard Wolf GmbH (Knittlingen, Germany)*, equipped with a 90$^{\circ }$ tip. The HSVs were captured with a spatial resolution of $256~\times ~256~\text {px}$ and a frame rate of $4,000~\text {fps}$. A total of 71 HSV recordings from stationary phonation were analyzed, comprising $N_{healthy} = 36$ recordings from healthy (#m: 16, #f: 20), $N_{paresis} = 26$ recordings from subjects with unilateral VF paresis (#m: 8, #f: 18), and $N_{polyp} = 8$ recordings from subjects with a unilateral VF polyp (#m: 4, #f: 4). All participants were asked to perform a sustained phonation of the vowel /æ/ at a comfortable pitch and loudness for at least $1~\text {s}$ during the examination. Additionally, two voice onsets (non-stationary phonation) were recorded from a single healthy subject (m, 30 yrs), who was asked to perform both a ’normal’ and a ’hard’ voice onset on the vowel /æ/.

### Laryngovibrogram

#### Segmentation of High-Speed Videos

The LVG representation introduced here requires reliable extraction of both the glottis and the VFs’ tissues from laryngeal HSVs. Our group recently developed a Deep Learning-based image segmentation procedure to fully automatically retrieve the glottal area and the VFs’ tissues from HSV recordings^85^. This approach achieves high segmentation accuracy comparable to that of manual expert segmentation, even for HSVs with lower image quality. The basic concept of this ANN-based segmentation procedure is briefly summarized below, as the LVG computation relies on its output.

**U-LSTM Neural Network** A deep convolutional ANN is used to automatically segment both the glottal area $A_{G}(t)$ and the VFs’ tissues $A_{VF_{l,r}}(t)$ from each frame *I*(*x*, *y*, *t*) of an HSV sequence with $t \in \{1,...,T\}$ subsequent frames. The segmentation procedure is based on the U-Net architecture introduced by Ronneberger et al.^88^. The U-Net is a widely used convolutional ANN from the encoder-decoder class, developed for single-image segmentation purposes, and is capable of extracting detailed image structures on a fine scale. Arabelle et al. refined the U-Net by adding Long Short-Term Memory (LSTM) cells to incorporate temporal information, resulting in the so-called U-LSTM^89^.

The U-LSTM implementation used in this work was specifically created for automatic glottis and VF segmentation in laryngeal HSVs and solves a 4-class segmentation problem (background, glottis, VF right, and VF left)^85^. It features a depth of five levels and is equipped with bi-directional LSTM cells in both the contracting and expanding paths. The LSTM cells process the sequence in both temporal directions^90^ to incorporate temporal information from the video. This bi-directional processing significantly improves the segmentation precision of laryngeal structures compared to the single-image segmentation approach of the U-Net^85^. The potential for such improvements has also been highlighted in recent studies by Nobel et al.^91^, Pedersen et al.^92^, and Dadras et al.^93^, which built on the U-LSTM approach to address various challenges in clinical voice diagnosis.

The U-LSTM used here was trained, validated, and tested on a dataset of 13,000 video frames from 130 HSV sequences, comprising recordings from both healthy and pathological subjects (e.g., muscle tension dysphonia, paresis, polyp, and carcinoma)^85^. Intense evaluation of segmentation congruency and accuracy demonstrated that, even in cases of low image quality, a diagnosis-independent overall segmentation precision in the range of manual expert segmentation or even higher could be achieved. However, though this dataset included a range of pathological cases, the generalization ability of the U-LSTM segmentation model to other laryngeal conditions was not explicitly evaluated. A detailed description of the U-LSTM-based segmentation approach can be found in the work of Fehling et al.^85^.

**Fine-tuning using user-in-the-loop** For this work, a user-in-the-loop approach was used to improve the U-LSTM’s segmentation with minimal user effort, ensuring a good and stable evaluation basis for the proposed approach. This additional step is, however, not a prerequisite for the presented method.

The proposed representation of VF dynamics depends on the reliable extraction of the glottis and the VFs’ tissue from the HSVs, thus accurate segmentation is crucial. Although the U-LSTM was specifically trained to extract both the glottis and the VFs, training was performed only on relatively short sequences of 100 HSV frames duration (25 ms) and exclusively on stationary phonations^85^. This increases the risk of segmentation artifacts especially in non-stationary HSV sequences, where non-stationarity may arise from non-stationary phonation or minor adjustments in laryngeal configuration. Since the ANN was trained on sequences with always - more or less - vibrating VFs, sequences without oscillating VFs are particularly prone to errors. For instance, in the pre-phonatory situation, the trachea as a ’structure unknown to the ANN’ may be visible between the VFs, potentially leading to incomplete segmentation of the glottis. Additionally, in some cases, irregular VF contours over time resulted in fluctuating vibration axes, affecting the stability of the extracted motion trajectories. While the U-LSTM has demonstrated high segmentation accuracy across various laryngeal conditions, its performance may be affected under extreme lighting variations or poor image quality - a known challenge for deep ANN-based approaches^94–97^. To avoid the need for time-consuming manual post-processing due to segmentation artifacts in such challenging HSV sequences, in this work, a user-in-the-loop approach was explicitly applied to fine-tune the pre-trained ANN for the specific recordings. A user interface was utilized to review and correct the segmentation results. The subsequent adaptation of the pre-trained U-LSTM to the task through re-training with the corrected segmentations took only a few seconds. This ’user-in-the-loop’ approach improved segmentation performance even for complex HSV images, while requiring minimal human intervention. Although a quantitative evaluation of these errors was not conducted, segmentation quality was visually assessed to ensure the necessary segmentation quality.

#### Process of LVG construction

**Fig. 3:**
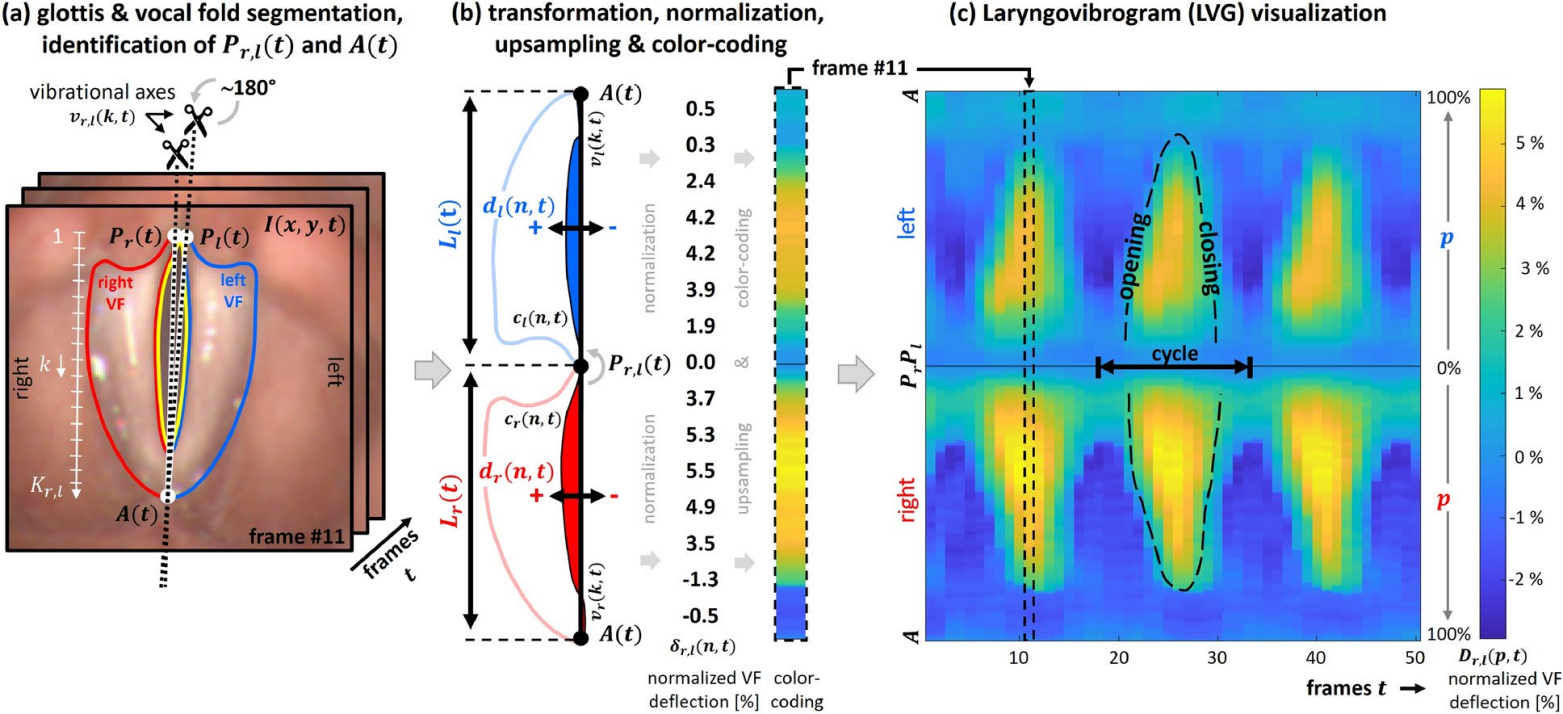
Computation of the LVG representation to visualize the laryngeal dynamics from high-speed videos. (**a**) Initial image processing step, which involves the segmentation of the glottis and the VFs, as well as the identification of the posterior points $P_{r,l}(t)$ and the anterior commissure *A*(*t*) in each frame *I*(*x*, *y*, *t*). These points $P_{r,l}(t)$ and *A*(*t*) are then used to define the individual vibrational axes $v_{r,l}(k,t)$ for each VF. (**b**) Contour-splitting and rotation of the left VF contour by approximately 180$^{\circ }$ counter-clockwise around the point $P_{r,l}(t)$. The VF deflections $d_{r,l}(n,t)$ are computed orthogonally to their respective vibrational axes $v_{r,l}(k,t)$. Subsequently, the deflections $d_{r,l}(n,t)$ are normalized to the individual VF lengths $L_{r,l}(t)$, resulting in the normalized VF deflections $\delta _{r,l}(n,t)$. The resulting deflections are upsampled to 256 equidistant points, translated into the relative positions *p*, and color-coded, yielding in one color strip per frame. (c) Concatenation of these color strips over time builds the LVG, where characteristic geometric patterns reflect the VFs’ vibrational behavior along their entire length. Positive deflections $D_{r,l}(p,t)$ indicate lateral VF movements, while a negative algebraic sign indicates a contra-lateral VF movement.

The Laryngovibrogram (LVG) introduced here aims to overcome the limitations of the PVG that result from its restriction to the region of the glottal opening by mapping the vibrational dynamics along the entire VF tissue length, enabling a more comprehensive analysis of the laryngeal dynamics. The construction of the LVG representation consists of three distinct steps: First, the initial segmentation of the VFs tissues, including the glottal area in between, is performed for each frame of an HSV recording. This segmentation serves as the basis for defining the individual vibrational axes of the VFs. Second, the VF deflections are computed with respect to the individual vibrational axes of the VFs, followed by normalization and color-coding of these normalized VF deflections. Third and finally, the LVG representation is constructed by concatenating these color strips, encoding the VFs’ spatiotemporal vibrational behavior along their entire length. The overall process of LVG construction is illustrated in Figure 3 and elaborated in more detail in the following.

**Step 1: Segmentation and identification of vibrational axes.** Initially, the VFs’ tissues and the glottal area are segmented using the U-LSTM. Based on the segmentation results, the points $P_{r,l}(t)$ and *A*(*t*) are determined for each HSV frame *I*(*x*, *y*, *t*). The points $P_{r,l}(t)$ approximate the position of the respective *Processus Vocalis*, marking the start of the VFs oscillatory part, while the point *A*(*t*) approximates the anterior commissure. Technically, $P_{r,l}(t)$ are defined in the segmentation results as the most dorsal points either shared between both VFs or between the respective VF and the segmented glottal area. The point *A*(*t*) is similarly defined as the most ventral contact point between the two VFs.

Non-stationary phonations, as well as normal VF vibrations, can cause the points $P_{r,l}(t)$ and *A*(*t*) to move slightly throughout an HSV sequence. To avoid any discontinuities in the latter LVG representation, a sliding window approach is applied with a window size of $150~\text {frames}$ and an overlap of $25~\text {frames}$ to interpolate the points over time. If the glottal area waveform (GAW) reaches zero at any point within a window, complete glottal closure is assumed, and the locations of points $P_r$ and $P_l$ are considered identical. Subsequently, the VF individual vibrational axes $v_{r,l}(k,t)$ are defined as a linear connection between the respective point $P_{r,l}(t)$ and the anterior commissure *A*(*t*) (see Fig. 3 (a)).

**Step 2: Computation of normalized VF deflections.** The LVG computation follows a similar principle to the PVG, but instead of focusing solely on the glottal area, it maps the entire VF tissue contours $c_{r,l}(n,t)$ between the points $P_{r,l}(t)$ and *A*(*t*), extending the analysis to include the full length of the VF. The left VF is virtually rotated by approximately 180$^{\circ }$ counter-clockwise around the dorsal VF endpoint $P_{l}(t)$, so both VFs are arranged alongside each other (Fig. 3 (a,b)). For each frame *t* and for all points $n \in \{1,2,...,N_{r,l}\}$ along the VF contour $c_{r,l}(n,t)$, the absolute distance $d_{r,l}(n,t)$ between the VF contour $c_{r,l}(n,t)$ and the respective vibrational axis $v_{r,l}(k,t)$ (with $k \in \{1,2,...,K_{r,l}\}$) is computed orthogonally (Fig. 3 (b)) using the $L_2$-norm. This corresponds to finding the minimum distance from the contour point to any point along the vibrational axis:

1 $$\begin{aligned} d_{r,l}(n,t) = \min \limits _{\kappa } \left\| v_{r,l}(\kappa ,t) - c_{r,l}(n,t) \right\| _2 \end{aligned}$$

with $\kappa \in \mathbb {R} \cap [1, K_{r,l}]$, where $v_{r,l}(\kappa ,t)$ represents the coordinates of the vibrational axis at $\kappa$. These absolute distances $d_{r,l}(n,t)$ are then normalized by the length $L_{r,l}(t)$ of the respective vibrational axis, yielding the normalized VF deflections $\delta _{r,l}(n,t)$:

2 $$\begin{aligned} \delta _{r,l}(n,t) = \frac{d_{r,l}(n,t)}{L_{r,l}(t)} \end{aligned}$$

These calculated $N_{r,l}$ deflections along the VF edges are upsampled to 256 equidistant points each, transformed into the relative positions *p*, and finally stored within a column vector $D_{r,l}(p,t)$, where the algebraic sign reflects the direction of the VF deflection. Positive values indicate a lateral VF deflection, while negative values denote a contralateral VF deflection. The normalized VF deflections $D_{r,l}(p,t)$ are then color-coded, resulting in one color stripe per HSV frame (Fig. 3 (b)).

**Step 3: Construction of the LVG.** Iterating the above steps over all frames of an HSV sequence and concatenating the normalized and color-coded deflections across time builds the LVG visualization (Fig. 3 (c)).

In the LVG, the vibrational behavior of the VFs is represented in a compact and normalized manner along the entire length of the VFs’ tissues, where spatiotemporal patterns encode vibrational properties over time. In this representation, the vibrational behavior of the left VF is displayed in the upper half, while the lower half of the LVG represents the right VF (Fig. 3 (c)). Each row in the LVG corresponds to a motion trajectory, describing the VF vibration at a specific VF position over time, while each column reflects the normalized VF deflections along the entire length of the VFs at a given point in time. Irregularities in VF vibration are indicated by variations in the spatiotemporal LVG geometry, while asymmetries in the vibrational behavior between the left and right VF can be assessed by comparing upper and lower halves of the LVG.

#### Quantitative LVG measures

The following quantitative LVG measures were used to evaluate the clinical applicability of the LVG, with a specific focus on the medial VF position ($p = 50\%$). These measures aim to provide an initial demonstration of the potential of an LVG-based analysis for assessing VF vibration, while a more detailed evaluation should be the focus of future studies.

**Relative normalized deflection** The relative vibration amplitude (RVA) is a frequently used parameter for evaluating the VFs’ vibrational behavior. It is defined as the cycle-wise difference between the minimum and maximum values of the motion trajectory, reflecting the relative VF deflection between its extreme oscillation states^27^. In the LVG, VF deflections are normalized to the length of the VFs. To highlight this distinction, the RVA computed from normalized VF deflections is referred to as the relative normalized deflection (RND). This measure can be calculated for each individual oscillation cycle $j \in \{1,2,...,J\}$, where *J* represents the total number of complete oscillation cycles, and $t_j^{start}$ and $t_j^{end}$ represent the start and end times of cycle *j*:

3 $$\begin{aligned} RND_{r,l}(p,j) = \max \limits _{{ t }} (D_{r,l}(p,t)) - \min \limits _{{ t }} (D_{r,l}(p,t)) ~~~~~~~~ \text { where }t \in [t_j^{start}, t_j^{end}] \end{aligned}$$

To describe the average magnitude of the VF deflection at VF position *p* over the entire HSV sequence, the average RND is calculated as:

4 $$\begin{aligned} \overline{RND}_{r,l}(p) = \frac{1}{J} \cdot \sum _{j=1}^{J}{RND_{r,l}(p,j)} \end{aligned}$$

**Shimmer** The LVG-based relative shimmer of the motion trajectory was used to evaluate the stability of the VF vibration amplitudes $A_j(p)$ throughout an HSV sequence. Within the LVG, the relative shimmer $shim_{r,l}(p)$ at VF position *p* is defined as:

5 $$\begin{aligned} shim_{r,l}(p) = \frac{ \frac{1}{J-1}\sum _{j=1}^{J-1}{ \left|A_j(p) - A_{j+1}(p) \right|} }{ \frac{1}{J} \sum _{j=1}^{J}{ A_j(p) } } \cdot 100 ~[\%] \end{aligned}$$

For a perfectly stable lateral vibrational behavior, the LVG-based relative shimmer for the sequence would be $shim_{r,l}(p) \equiv 0$.

Shimmer and jitter have been established as key perturbation measures in acoustic voice analysis, and their use has been extended to high-speed imaging of VF vibrations (e.g., Inwald et al.^98^, Kist et al.^99^, Pietruszewska et al.^100^, Schlegel et al.^101^, Yan et al.^49^). By applying these measures to the LVG, a direct comparison of vibratory irregularities across different analysis modalities is enabled while consistency with existing literature is maintained, facilitating clinical interpretability and supporting the validation of LVG-based assessments in future research.

**Jitter** The LVG-based relative jitter of the motion trajectory was used to evaluate the temporal stability of VF vibration throughout an HSV sequence. Within the LVG, the relative jitter $jit_{r,l}(p)$ at VF position *p* is defined as:

6 $$\begin{aligned} jit_{r,l}(p) = \frac{ \frac{1}{J-1}\sum _{j=1}^{J-1}{ \left|T_j(p) - T_{j+1}(p) \right|} }{ \frac{1}{J} \sum _{j=1}^{J}{T_j(p)} } \cdot 100 ~[\%] \end{aligned}$$

where $T_j(p)$ represents the duration of the *j*-th oscillation cycle. For a perfectly stable temporal vibrational behavior, the LVG-based relative jitter would be $jit_{r,l}(p) \equiv 0$.

**Lateral vibration symmetry** The Quotient of the normalized deflections of both VFs, denoted as $Q_{RND}$, was used to evaluate the symmetry of lateral VF vibration amplitudes. For a given VF position *p* and for oscillation cycle *j* within the LVG, the $Q_{RND}(p,j)$ is defined as:

7 $$\begin{aligned} Q_{RND}(p,j) = \frac{min(RND_{r}(p,j), RND_{l}(p,j))}{max(RND_{r}(p,j), RND_{l}(p,j))} \end{aligned}$$

The average $\overline{Q}_{RND}$ at VF position *p* over an entire HSV sequence is defined as:

8 $$\begin{aligned} \overline{Q}_{RND}(p) = \frac{1}{J}\sum _{j=1}^{J}{ Q_{RND}(p,j) } \end{aligned}$$

Because the $Q_{RND}$ does not depend on which VF is affected, it provides a metric for assessing group-specific differences in lateral VF vibration amplitude symmetry. In the case of perfectly overall symmetrical vibrational behavior, with identical RNDs for both VFs, the quotient would be $Q_{RND}\equiv 1$.

**Lateral phase synchronity** The lateral phase difference $\Delta \overline{\Theta }(p)$ was used as a clinically interpretable measure of the synchronity between the vibrations of both VFs’. The phase signals $\phi _{r,l}(p,t)$ for the evaluated motion trajectories were determined using a complex wavelet analysis as described by Unger et al.^35^. For an entire HSV sequence consisting of *T* subsequent frames, the lateral phase difference $\Delta \overline{\Theta }(p)$ is computed as

9 $$\begin{aligned} \Delta \overline{\Theta }(p) = | \frac{1}{T} \sum _{t=1}^{T}{ \arg [ \exp ( i \phi _{l}(p,t) - i \phi _{r}(p,t) ) ] |} \end{aligned}$$

with the phase angles transformed to the complex unit circle to ensure that all lateral phase differences remain within the range of $-\pi$ to $\pi$. In the case of perfectly synchronous vibrational behavior of both VFs, the lateral phase difference would be $\Delta \overline{\Theta }(p) \equiv 0$. However, any asynchrony may indicate pathological conditions, highlighting the clinical significance of measuring lateral phase differences along the vibrating VF tissue^35^. To maintain consistency with the average lateral phase difference Unger et al. has computed from PVG^35^, the average lateral phase difference within the glottal opening region of the LVG is considered and denoted as $\Delta \overline{\Theta }_{op}$. Additionally, the average lateral phase difference computed from the entire LVG is denoted as $\Delta \overline{\Theta }_{LVG}$.

### Clinical LVG application

The differences in the representation of the laryngeal dynamics between the introduced LVG and the previous PVG are illustrated by comparing two typical clinical examples, as discussed in section (a) Comparing PVG and LVG. The introduced measures are applied not only to analyze these differences but also to assess clinical LVG application through intra- and inter-individual comparisons, as well as non-stationary phonation, as covered in sections (b) LVG analysis of repeated measures within a healthy subject, (c) Cross-sectional comparison of LVG analysis between clinical groups, and (d) LVG analysis of voice onset.

#### Comparing PVG and LVG

Exemplarily, the PVGs and LVGs constructed from two clinical HSV recordings are assessed to compare overall differences between both representations. The investigated HSVs are from a healthy subject (m, 25 yrs) and a patient with a unilateral VF polyp on the right VF (m, 25 yrs). For comparison purposes, the VF deflections derived from the glottal contour within a PVG are normalized relative to the length of the glottal axis. This normalization ensures that the PVGs are as comparable as possible across different individuals.

#### LVG analysis of repeated measures within a healthy subject

Intra-individual comparability of the LVG representation was investigated using three repeated recordings from the same healthy subject (m, 27 yrs) performing sustained phonations of the vowel /æ/ at a comfortable pitch and loudness. The vibrational behavior was qualitatively assessed using the LVG representation to evaluate the overall representation of the vibrational behavior, and quantitatively analyzed using the previously defined measures.

#### Cross-sectional comparison of LVG analysis between clinical groups

To gain an initial understanding of different clinical groups, LVGs and the corresponding PVGs were computed for a total of 66 HSVs comprising recordings from healthy subjects ($N_{healthy} = 32$, #m: 12, #f: 20) and pathological subjects ($N_{paresis} = 26$, #m: 8, #f: 18; $N_{polyp} = 8$, #m: 4, #f: 4). The RND was computed for each individual oscillation cycle and for both VFs, at the medial VF position for LVG and the medial glottis position for PVG ($p = 50\%$). To avoid any bias caused by the varying number of cycles per sequence, which results from the subject’s individual fundamental frequency, only the averaged parameters per HSV sequence were considered.

A group of healthy females and males was analyzed to identify potential sex-related variations in the LVG-based measures. Subsequently, the three clinical groups ’healthy’, ’paresis’, and ’polyp’ were examined to establish clinical reference data for the LVG-based measures for the first time. In this context, the group ’healthy’ included all female and male subjects from the previous analysis of potential sex-related variations in LVG-based measures. Additionally, all parameters were also computed for the PVG to provide reference values for descriptive comparison. To further investigate differences between PVG- and LVG-based measures, the variability in the location of the medial PVG trajectory was assessed relative to VF length, as used for LVG construction, across both VFs and all three clinical groups. To investigate the magnitude of effect for group comparisons across the clinical groups, the absolute values of Cliff’s Delta were assessed, with all investigated parameters pooled separately for PVG and LVG.

Shapiro-Wilk tests were used to assess the normal distribution of the investigated parameters in healthy females and males. Following that, Mann-Whitney-U-tests were performed to check for significant differences between these groups. To assess potential differences in variance between PVG- and LVG-based measures, four Levene tests (one per investigated parameter) were conducted for the healthy subjects, comparing the PVG-based values, pooled across healthy males and females, to the respective LVG-based values for each parameter. Pearson’s Linear Correlation Coefficient was used to evaluate whether the RND correlates with the fundamental frequency $F_0$ in healthy subjects. For the pathological groups ’paresis’ and ’polyp’, a Pearson Chi-square test was applied to determine whether the VF side with the lower RND corresponded to the affected VF side, as indicated in the patients’ records. Shapiro-Wilk tests were used to assess whether the investigated parameters in the clinical groups followed a normal distribution. Kruskal-Wallis tests were performed to compare the distributions across the three clinical groups, followed by paired Mann-Whitney-U posthoc-tests to identify significant parameter differences. The same statistical analyses were also conducted for the PVG-based parameters as part of the overall assessment. In all tests, the significance level was set to $\alpha = 0.05$, and p-values were adjusted using the Benjamini-Hochberg procedure where applicable and are reported as $p^*$.

#### LVG analysis of voice onset

Finally, the suitability of the LVG for reliably representing complex non-stationary phonation paradigms was assessed using HSV recordings from two voice onsets of a single healthy subject. It was examined whether the LVG can reliably represent the VF dynamics thoughout such a complex transition process, which includes VF adduction, the onset of VF vibration, and sustained phonation. Additionally, the transition from the pre-phonatory situation to the onset of VF vibration was quantified by tracking the VF opening angle $\Gamma (t)$ over time, which is defined here as the angle enclosed by the two VFs’ individual vibrational axes. To further explore the potential of an LVG-based analysis in providing new insights into laryngeal physiology, a comparison between a ’normal’ and a ’hard’ voice onset was performed on the same subject (m, 30 yrs). The hardness of the voice onset is determined by the time derivative of the VF opening angle, which corresponds to the angular velocity of the VFs adduction movement. To quantitatively evaluate the VF adduction movement, the average angular velocity ($\gamma$) was calculated using linear regression over a time interval immediately preceding the voice onset (start of oscillation). Specifically, a time window of $1000~\text {frames}(250\text {ms})$ was used in this work.

## Results

Different aspects of how the LVG represents the VFs’ vibrational behavior were evaluated to investigate whether the proposed LVG could overcome the limitations of the PVG and enhance the interpretation of VF dynamics. Additionally, it was examined whether the LVG representation facilitates the interpretational power and provides a more robust quantitative analysis to support clinical applications. To evaluate its applicability for the analysis of laryngeal dynamics, the LVG was evaluated on common clinical scenarios. Specifically, it was applied to both intra- and inter-individual comparisons of stationary HSV recordings, and its potential for analyzing non-stationary phonations was explored.

### Comparing PVG and LVG

**Fig. 4:**
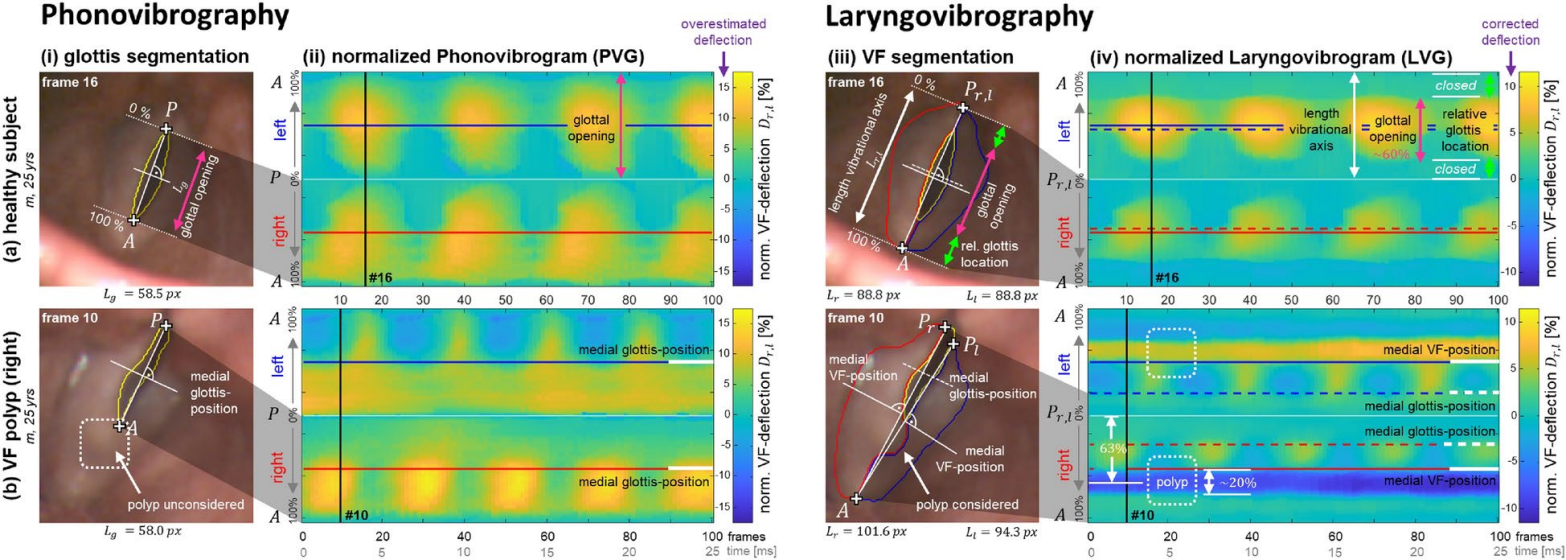
Comparison of Phonovibrogram (PVG) and Laryngovibrogram (LVG) representation of the VF’s vibrational behavior exemplarily demonstrated using two clinical HSV recordings. (**a**) Limitation 1: ’Unknown range and location of glottal opening’, demonstrated on a healthy male subject (25 yrs). (**b**) Limitation 2: ’No consideration of the individual vibrational axes’, demonstrated on a male patient (25 yrs) with a unilateral VF polyp on the right VF. (i, ii) The PVG visualizes the VFs’ vibrational dynamics only in the range of the glottal opening. (iii, iv) The LVG visualizes the VF dynamics along the entire VF length, providing additional information on the range and the location of the glottal opening as well as on any organic lesions that may be present on the VFs. Solid lines inside the panels indicate the medial position in both the PVG and LVG, corresponding to the medial glottis position in the PVG (i, ii) and the medial VF position in the LVG (iii, iv). The dashed lines in the LVG provide a visual reference for the medial glottis position.

The first analysis of this work aimed to evaluate whether the LVG can overcome the limitations of the PVG by providing a more comprehensive representation of the VFs’ vibrational behavior. Additionally, this analysis explored the overall differences between the two representations. Figure 4 shows the VFs’ vibrational behavior from two individual subjects, using both the PVG and LVG representations. The first limitation of the PVG, ’Unknown range and location of glottal opening’, is illustrated in panels (a) on a healthy male subject (25 yrs), while the second limitation, ’No consideration of the individual vibrational axes’, is illustrated in panels (b) on a 25-year-old male patient with a unilateral VF polyp on the right VF. For each subject, a frame with maximal glottal opening demonstrates the concrete construction of (i) the PVG and (iii) the LVG. While the construction of the PVG relies on the glottal opening, from which the VFs’ edges are deduced, the construction of the LVG directly considers the segmented VFs’ tissues themselves. Panels (ii) show the resulting PVGs, while panels (iv) show the corresponding LVGs constructed from the same recordings. The medial positions of the glottis and the VFs are indicated in the frames as well as in the corresponding PVG and LVG representations.

The healthy subject shown in Figure 4 (a) exhibits a regular, symmetric, and synchronous vibrational behavior, which is clearly visible in both the PVG and LVG representations (Fig. 4 (a)(ii) & (a)(iv)). However, since the extracted glottal area cannot provide information on the relative range of the glottal opening or the location of the glottis itself (Limitation 1), the PVG maps the VFs’ vibrational behavior from the range of glottal opening relative to the glottal symmetry axis (Fig. 4 (a)(i, ii)). In contrast, by additionally extracting the visible VFs’ tissues, the LVG can provide insights into both the range and the relative location of the glottal opening (Fig. 4 (a)(iii) & (iv)). For the healthy subject presented here, the LVG shows that the VFs open at their center, with an extent of about 60% of their length (Fig. 4 (a)(iv)). By comparison, in the PVG, the lateral VF deflections can only be normalized to the length of the glottal symmetry axis to improve the comparability of different HSV sequences. However, in this case, the normalized VF deflections in the PVG are estimated to be too high, which becomes clear when compared with the normalized VF deflections in the LVG. Furthermore, this HSV sequence demonstrates that the two vibrational axes coincide when both VFs have permanent contact at their dorsal ends.

By contrast, the VFs of the subject shown in Figure 4(b) do not make dorsal contact. As a result, the two vibrational axes differ from each other (Fig. 4(b)(iii)) and do not align with the glottal symmetry axis (Limitation 2). A prerequisite for obtaining reliable results from quantitative inter-individual comparisons is that the vibrational behavior is consistently evaluated at identical VF positions. Frequently, the medial VF position is in the focus of clinical evaluation, as the largest VF amplitude is usually observed in the middle of the membraneous portion of the VFs^19,102,103^. However, estimation of the medial VF position is not possible based solely from the extracted glottal area. Consequently, in the PVG, the medial glottis position is assumed to approximate the medial VF position. And while the medial glottis position and the medial VF position align quite well in cases where the glottal opening is located medially (Fig. 4(a)(iii) & (iv), healthy subject), the example with a polyp in Figure 4(b)(ii) & (b)(iv) demonstrates that when the glottal opening is limited or obstructed, substantial discrepancies may arise, and the medial glottis position may no longer coincide with the medial VF position. Here, the polyp at the ventral to the medial part of the right VF hinders the opening of the VFs at their ventral part throughout the entire HSV sequence. As a result, the polyp itself is not incorporated into the PVG visualization. Moreover, the PVG captures the vibrational behavior of approximately only half of the VFs’ entire length. This leads to an incorrect estimation of the medial VF position in the PVG. The LVG, on the other hand, includes information about the polyp, such as its location, size, lateralization, and orientation. It thus enables a quantitative assessment of the polyp’s impact on the vibrational behavior. In the case presented here, the polyp has an extent of about 25% of the VF length and is localized in the anterior to medial third, around the 63% position on the right VF. The LVG’s coloration further indicates that the polyp causes a local and static indentation on the opposite VF (Fig. 4(b)(iv), dashed box).

Overall, the LVG offers a representation of the vibrational behavior along the entire VF length, including regions that remain closed during oscillation. By overcoming the PVG’s limitation of focusing solely on the glottal area, the LVG provides a more comprehensive view of VF dynamics. As the vibrational behavior in the LVG maintains a geometric pattern quite similar to that of the PVG, it facilitates easier interpretation for clinicians who are already familiar with the PVG.

### LVG analysis of repeated measures within a healthy subject

**Fig. 5:**
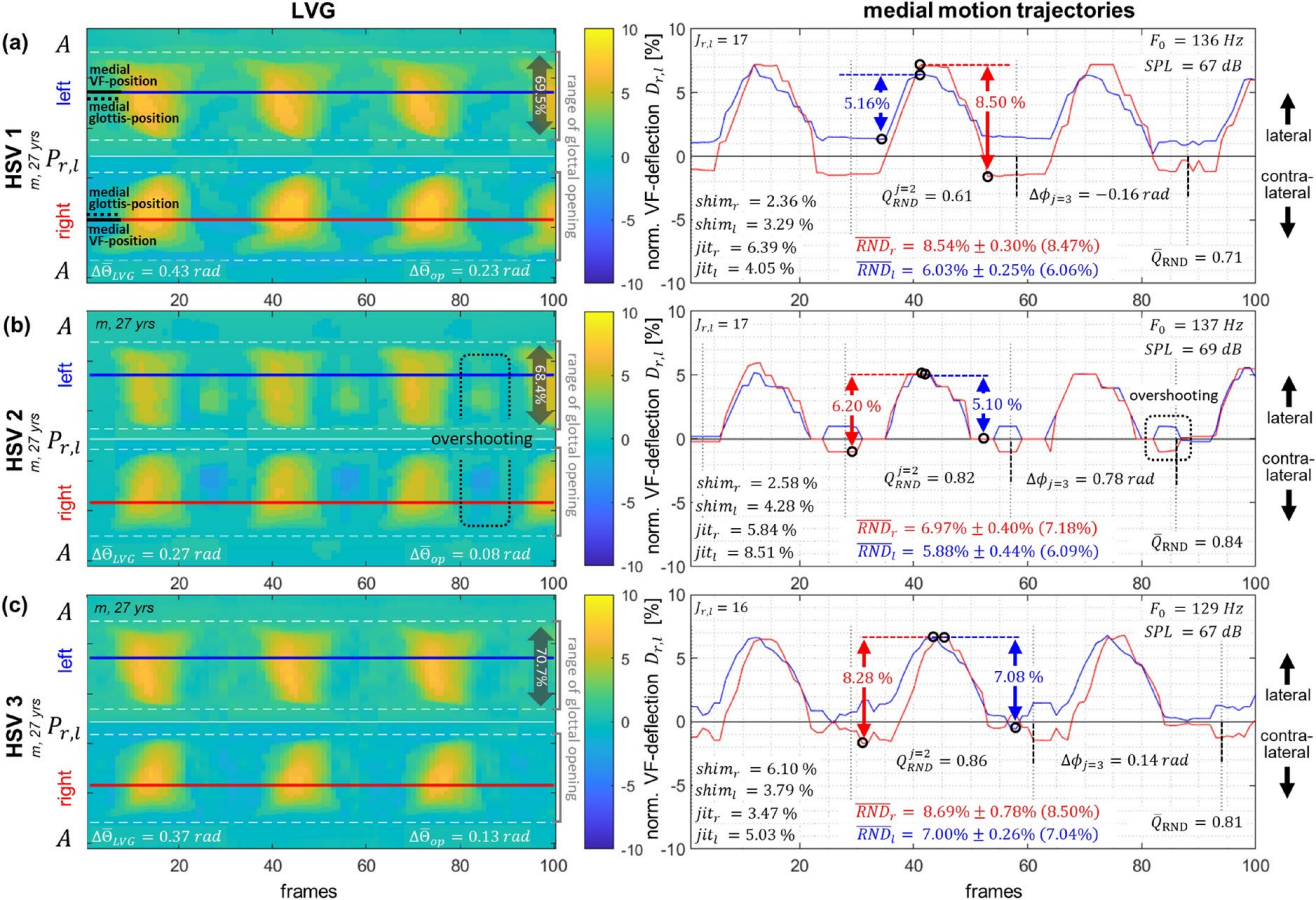
LVGs and the motion trajectories at the medial VF position, derived from three HSV recordings of a single healthy subject (m, 27 yrs) who was repeatedly recorded during sustained phonation. Each sequence comprises 500 frames, but only 100 frames are shown for visualization purposes. $\Delta \overline{\Theta }_{op}$ inside the LVG visualization indicates the average lateral phase difference in the range of the glottal opening, while $\Delta \overline{\Theta }_{LVG}$ indicates the average lateral phase difference along the entire VFs lengths. In the panels showing the medial motion trajectories, the lateral phase difference, the *RND*, and the $Q_{RND}$ are indicated exemplarily for one cycle. Additionally, the average *RND* for both VFs individually $\overline{RND}_{r,l}$, the shimmer $shim_{r,l}$, the jitter $jit_{r,l}$, the average lateral amplitude symmetry $\overline{Q}_{RND}$, the fundamental frequency $F_0$, and the sound pressure level *SPL* are provided inside the trajectory panels.

Intra-individual comparability is crucial in clinical routine, particularly for tracking therapy outcomes. Therefore, this analysis focused on assessing LVG-based intra-individual comparability by investigating repeated recordings of sustained phonation from a single healthy subject. Figure 5 shows the LVGs and the motion trajectories at the medial VF positions for three HSV recordings of the same healthy subject (m, 27 yrs). For each recording, the subject was asked to perform a sustained phonation of the vowel /æ/ for at least 2 seconds at a comfortable pitch and loudness. The three analyzed sequences each comprise 500 frames of the recorded HSVs, but for better visualization, only the first 100 frames are shown. The corresponding LVGs are shown on the left, with detailed views of the motion trajectories from the medial VF positions displayed on the right (Fig. 5). The LVGs reveal a good overall agreement of the vibrational behavior across all three HSV sequences, with comparable LVG geometry and a similar extent of glottal opening, approximately 70% of the VFs length in each (white dashed lines and gray arrows, Fig. 5). All sequences show regular, symmetrical, and synchronous VF vibrations. As the fundamental frequency in *HSV 3* (129 Hz) is slightly lower compared to the other two sequences (136 Hz and 137 Hz, respectively), only 3 instead of 3.5 oscillation cycles are visible in the respective LVG. Additionally, the LVG coloration of *HSV 2* indicates that the normalized VF deflection is slightly reduced compared to both of the other sequences.

To provide a detailed view of the vibrational behavior, the right panels of Figure 5 visualize the normalized VF deflections at the medial VF position. Positive values in these motion trajectories indicate lateral deflection of the respective VF, while negative values indicate a contra-lateral deflection. In cases where both vibrational axes are identical, the motion trajectories during the closed phase appear symmetrical, but with opposite algebraic signs. If VF contact during the closed phase occurs directly on the vibrational axis, the normalized deflection during the closed phase is 0% (see *HSV 2*). In contrast, laterally shifted VF contact during the closed phase is indicated by symmetrical offsets in the motion trajectories for both VFs, but with opposite algebraic signs (see *HSV 1* & *HSV 3*). The RND is indicated exemplarily for one cycle of each sequence (red and blue arrows). Additionally, the average RNDs with mean, standard deviations and median provided in brackets are given for all analyzed oscillation cycles inside the respective panels for both VFs independently.

The right and left VF motion trajectories show good conformity within the visualized oscillation cycles across all three sequences. All three HSV sequences for this subject showed $\overline{RND}_{r,l}$ values within approximately the same range, with average RNDs between $5.88 \%$ to $8.69 \%$. In each case, the left VF exhibited slightly reduced $\overline{RND}_{l}$ compared to the right VF’s $\overline{RND}_{r}$. Moreover, compared to the other sequences, *HSV 2* shows overall slightly reduced RNDs. RND variation remained consistent with high lateral amplitude stability for all three sequences, as indicated by shimmer values ranging from $2.36 \%$ to $6.10 \%$.

Sometimes both VFs oscillate during the closed phase together beyond their vibrational axes toward one VF. This can, in general, be either due to the endoscopic viewing angle onto the VFs’ vibration plane or the presence of some pathologies and leads to a characteristic pattern in the LVG as well as in the individual motion trajectories (dashed-boxes in Fig. 5 (b)). In the LVG visualization, this behavior becomes evident through regions with similar normalized VF amplitudes but opposite algebraic signs, indicating the reverse directions of the VF individual deflections (’+’ lateral deflection, ’-’ contra-lateral deflection). This ’overshooting’ behavior might negatively affect the $RND_{r,l}$ values, as demonstrated in the sequence *HSV 2*. In this case, the trajectories show a very high agreement during the opened phase, but the VFs oscillate together beyond the shared vibrational axis during the closed phase. This leads to a higher $\overline{RND}_{r}$ for the right VF, which, in *HSV 1*, is on average approximately 20% higher than the left VF’s $\overline{RND}_{l}$. Similarly, the $Q_{RND}$ is affected by the overshooting behavior, as indicated by a reduced $Q_{RND} = 0.82$ for the depicted cycle of *HSV 2*. However, with the $\overline{Q}_{RND}$ ranging from 0.71 to 0.84, all three HSV recordings showed high lateral amplitude symmetry throughout the entrire sequence.

Jitter measurements ranging from $3.47 \%$ to $8.51 \%$, indicated only slight variations in temporal stability across all sequences and confirmed the impression of regular vibrational behavior. Furthermore, a lateral phase difference between the right and left VFs is visible in the medial trajectories, particularly for *HSV 1* and *HSV 3* (Fig. 5, trajectory panels). While the trajectories of *HSV 2* appear to have only minimal phase shift, the lateral phase difference for the depicted cycle is by 0.78 *rad* considerably greater than that observed for the depicted cycles from *HSV 1* and *HSV 3* ($-0.16~rad$ and 0.15 *rad*, respectively). This suggests that the ’overshooting’ behavior affects not only the vibratory amplitudes but also the phase relationship between the two VFs. The average lateral phase difference $\Delta \overline{\Theta }_{op}$ was additionally computed analogously to Unger et al.^35^ for the region of the glottal opening, yielding values between 0.08 *rad* and 0.23 *rad*. And as the LVG allows for an analysis along the entire length of the VFs, the average lateral phase difference $\Delta \overline{\Theta }_{LVG}$ was also computed for all trajectories across the entire VF length. By ranging from 0.27 *rad* to 0.43 *rad*, $\Delta \overline{\Theta }_{LVG}$ consistently exceeded $\Delta \overline{\Theta }_{op}$ in all three recordings.

### Cross-sectional comparison of LVG analysis between clinical groups

**Fig. 6:**
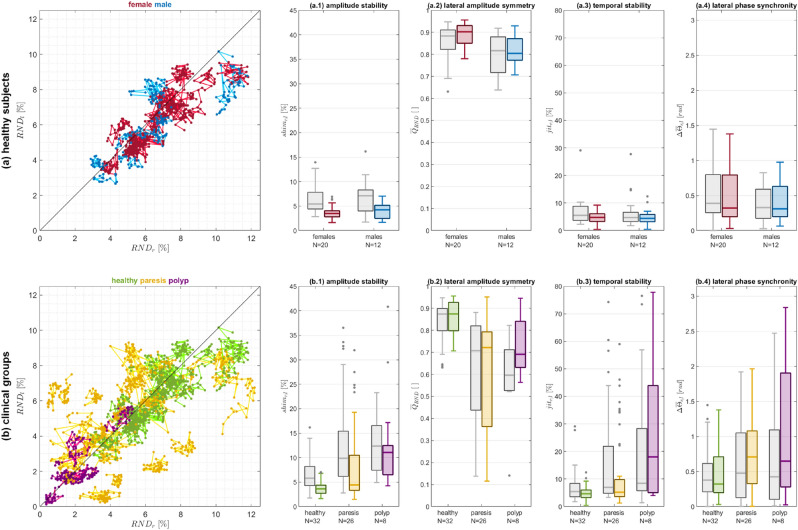
Evaluation of the VFs’ vibrational behavior concerning magnitude of VF deflection, amplitude stability, lateral amplitude symmetry, temporal stability, and lateral phase synchronity. (**a**) Healthy subjects subdivided by sex: ’female’ ($N = 20$, red), ’male’ ($N = 12$, blue). (**b**) Three clinical groups ’healthy’ ($N = 32$, green), ’paresis’ ($N = 26$, yellow), and ’polyp’ ($N = 8$, purple). Scatterplots in (**a**) and (**b**) visualize the $RND_r$ vs. $RND_l$ at medial VF position for each individual oscillation cycle of the investigated HSV sequences with the single sequences indicated by inter-connections. In the case of perfect symmetrical RNDs for both VFs of the considered cycle, the scatter point lies on the bisectrix (black line). The boxplots in (a.1)-(b.4) provide a closer look at different aspects of the VFs’ vibrational behavior with ’-’ indicating the median. Gray boxplots represent the PVG-based values, serving as a reference. All parameters are averaged per HSV sequence. *N* provides the number of HSV sequences per group. (a.1, b.1) Amplitude stability: Stability of the VF vibration amplitudes throughout the HSV sequence as measured by the relative shimmer *shim*. (a.2, b.2) Lateral amplitude symmetry: Symmetry in the vibrational behavior of both VFs as measured by the quotient $\overline{Q}_{RND}$. (a.3, b.3) Temporal stability: Temporal stability of the VFs’ vibrational behavior throughout the HSV sequence as measured by the relative jitter *jit*. (a.4, b.4) Lateral phase synchronity: Synchronity in the vibrational behavior of both VFs as measured by the mean lateral phase difference $\Delta \overline{\Theta }$. Detailed group-averaged results for all measures are provided in Table 1.

Inter-individual comparisons and clinical interpretation require reference data. This analysis aimed to provide an initial understanding of LVG-based measures across different clinical groups, taking into account both sex-related variations and pathological conditions. Therefore, LVGs and PVGs were computed for a total of 66 HSVs from both healthy and pathological subjects (unilateral VF polyps and unilateral VF paresis). Both LVGs and PVGs were evaluated based on the VFs’ vibrational behavior at their medial position using the introduced measures. Figure 6 presents the results of the analyses concerning the magnitude of VF deflection, amplitude stability, lateral amplitude symmetry, temporal stability, and lateral phase synchronity, which are shown for healthy individuals divided by sex (a.1 - a.4) and for the clinical groups (b.1 - b.4). In all panels, boxplots visualize the distribution of each parameter for the respective group, with the median indicated by ’-’. Gray boxplots represent the PVG-based values, serving as a reference. The group-averaged results for the investigated measures are provided in Table 1.

At first, a set of healthy females (#f: 20) and males (#m: 12) was investigated for the presence of sex-related variation in RND. The scatterplot in Figure 6 (a) provides a first impression of how the RNDs computed from the LVGs behave for healthy subjects in terms of their values and their variability. It visualizes the relationship between $RND_r$ and $RND_l$ at the medial VF positions, where each scatter point represents the pair $(RND_r(p=50\%,j), RND_l(p=50\%,j))$ for an individual oscillation cycle *j*, with connected scatter points indicating subsequent oscillation cycles of the same HSV sequence. The females are color-coded red, and the males blue. In cases where VF vibrations have perfectly symmetric VF amplitudes for the considered cycle, the scatter points are located directly on the bisectrix, and any inter-cycle variation of the RND is mapped along the bisectrix. Males and females show relatively similar distributions with comparable average RND values for the right as well as for the left VF ($\overline{RND}_{r,l}$), although the males exhibit in both VFs slightly more variability. However, a Mann-Whitney U-test indicated that the differences in RND between sexes are not statistically significant for either the right VF ($p = 0.83$, $\alpha = 0.05$) or the left VF ($p = 0.86$, $\alpha = 0.05$), suggesting that the investigated females and males come from a continuous distribution with equal medians, allowing for analyses regardless of sex. Furthermore, a weak, though statistically not significant negative correlation was found between the $RND_{r,l}$ and the fundamental frequency (Pearson’s Linear Correlation Coefficient $\rho = -0.21$, $p = 0.10$, $\alpha = 0.05$). In addition to the RND itself, amplitude stability, lateral amplitude symmetry, temporal stability, and lateral phase synchronity were analyzed for both PVG and LVG, where the PVGs serve as a comparative baseline.

Figure 6 (a.1) shows the relative shimmer *shim* as a measure of amplitude stability of VF vibration for healthy subjects separated by sex. Both male and female subjects exhibited similar LVG-based shimmer without any statistically significant differences ($p = 0.22$, $\alpha = 0.05$), indicating comparable amplitude stability in VF vibration across the sexes. As a measure of lateral amplitude symmetry, Figure 6 (a.2) illustrates the $\overline{Q}_{RND}$ for the healthy subjects by sex. Male subjects demonstrated a lower average LVG-based $\overline{Q}_{RND}$ compared to female subjects, with statistically significant differences in lateral amplitude symmetry between the sexes ($p = 4.49 \cdot 10^{-3}$, $\alpha = 0.05$). In terms of temporal stability, Figure 6 (a.3) presents the relative jitter *jit* for healthy subjects by sex. Regardless of their sex, the healthy subjects exhibited comparable temporal stability in VF vibration as represented by the LVG without any statistically significant differences ($p = 0.54$, $\alpha = 0.05$), indicating consistent temporal stability across the sexes. Finally, lateral phase synchronity was evaluated using the average lateral phase difference $\Delta \overline{\Theta }$. Figure 6 (a.4) shows that LVG-based phase differences were within a comparable range for both sexes, with no statistically significant differences ($p = 0.79$, $\alpha = 0.05$), indicating similar levels of lateral phase synchronity between males and females. To complement these findings, Table 1 provides the respective values for the corresponding PVG-based measures. While the PVG-based measures follow a similar pattern, some differences in variability and central tendency can be observed. To further investigate these differences, variance analyses between the PVG- and LVG-based measures were conducted using Levene tests in the healthy subjects across all four investigated parameters. The tests indicated statistically significant variance differences between PVG- and LVG-based values for the relative shimmer *shim* ($p = 2.5 \cdot 10^{-7}$, $\alpha = 0.05$) and the relative jitter *jit* ($p = 9.09 \cdot 10^{-3}$, $\alpha = 0.05$). In contrast, no significant variance differences were observed for the lateral amplitude symmetry $\overline{Q}_{RND}$ ($p = 0.27$, $\alpha = 0.05$) or the lateral phase difference $\Delta \overline{\Theta }$ ($p = 0.86$, $\alpha = 0.05$).

Since in the healthy subjects previously no statistically significant differences in terms of their RND-values were identified between both sexes, the following analyses analyzed the three clinical groups ’healthy’ ($N_{healthy} = 32$), ’paresis’ ($N_{paresis} = 26$), and ’polyp’ ($N_{polyp} = 8$) without regard of sex. Here, the healthy group comprised male and female subjects from the previous analysis on potential sex-related variations in the LVG-based measures. To further investigate differences between PVG- and LVG-based measures due to their methodological representation, the variability in the location of the medial PVG trajectory was analyzed relative to VF length, as used for LVG construction. In contrast to the LVG where the medial VF position ($p = 50\%$) is by definition always represented by the medial trajectory, the location of the PVG-based medial trajectory position along the VF varied across clinical groups. On average, the medial PVG trajectory was located at $42.35\% \pm 9.56\%~(42.19\%)$ of the VF length for healthy subjects, at $43.28\% \pm 8.85\%~(43.75\%)$ for patients with unilateral VF paresis, and at $45.58\% \pm 13.69\%~(45.12\%)$ for patients with unilateral VF polyps. For a visual representation of these differences, see Supplementary Figure.

The scatterplot in Figure 6 (b) shows the relation between $RND_r$ and $RND_l$ for all cycles of the investigated HSV sequences. Both the paretic subjects and the subjects with polyp show reduced average RNDs ($\overline{RND}_{r,l}$) when compared to the healthy subjects. The paretic subjects exhibit high variability between individual sequences (inter-sequential), but within individual sequences (intra-sequential), RND varies only slightly in most cases. The few paretic cases with high intra-sequential variability are associated with atonic paresis (RND variation is plausible according to the respective HSV sequence; detailed results not shown). For the polyp group, the average RND is reduced compared to both paretic and healthy subjects, with RND variation bein even slightly lower than normal RND variation. A Pearson Chi-square test of independence showed no significant relationship between the VF side with lower RND and the affected VF side as stated in patient records, for neither the paretic group ($\chi ^2(1, N = 26) = 0.45$, $p = 0.50$) nor the polyp group ($\chi ^2(1, N = 8) = 0.53$, $p = 0.47$). Due to the limited availability of HSV recordings from pathologic subjects, no distinction was made between the affected VF side.

In Figure 6 (b.1), the relative shimmer *shim* is presented for the three clinical groups ’healthy’, ’paresis’, and ’polyp’. Compared to the healthy subjects, both pathologic groups show an increased average relative shimmer, with larger standard deviations and a few outliers in each group. The Kruskal-Wallis test ($p^* = 2.1 \cdot 10^{-8}$, $\alpha = 0.05$) indicated a statistically significant difference between healthy and paretic subjects ($p^* = 2.62 \cdot 10^{-3}$), as well as between healthy subjects and those with polyps ($p^* = 1.46 \cdot 10^{-6}$). Additionally, a significant difference was found between the paresis and polyp groups ($p^* = 9.83 \cdot 10^{-3}$). These results indicate that any of the investigated pathologies affect the lateral VF vibration amplitude stability, whereas the investigated pathologies influence amplitude stability differently. Similarly, Figure 6 (b.2) visualizes the $\overline{Q}_{RND}$ for the three clinical groups ’healthy’, ’paresis’, and ’polyp’. Compared to the healthy subjects, the $\overline{Q}_{RND}$ is considerably reduced for both the pareses and the polyps. The Kruskal-Wallis test ($p^* = 2.11 \cdot 10^{-5}$, $\alpha = 0.05$) indicated a statistically significant change in the symmetry measure $\overline{Q}_{RND}$ between the clinical groups. Benjamini-Hochberg corrected post-hoc Mann-Whitney-U-tests confirmed a significant difference between the healthy and the paretic subjects ($p^*_{healthy,paresis} = 7.35 \cdot 10^{-5}$, $\alpha = 0.05$), while no significant difference was found between the healthy and the polyp group ($p^*_{healthy,polyp} = 0.073$, $\alpha = 0.05$) or between the two pathological groups ($p^*_{paresis,polyp} = 0.80$, $\alpha = 0.05$). These results indicate that lateral amplitude symmetry is significantly affected in paresis but not in polyp patients when compared to the healthy group, while no significant differences are observed between the two pathologic groups. In contrast to the amplitude-related measures, Figure 6 (b.3) shows the relative jitter *jit* for the three clinical groups ’healthy’, ’paresis’, and ’polyp’ measuring temporal stability. All three groups exhibit different average relative jitter values and variabilities. The Kruskal-Wallis test ($p^* = 3.15 \cdot 10^{-4}$, $\alpha = 0.05$) indicated a statistically significant difference between the three groups for the temporal stability. Benjamini-Hochberg corrected post-hoc Mann-Whitney-U-tests confirmed a significant difference between the healthy and polyp groups ($p^*_{healthy,polyp} = 5.68 \cdot 10^{-4}$, $\alpha = 0.05$) as well as between the paresis and polyp groups ($p^*_{paresis,polyp} = 3.53 \cdot 10^{-2}$, $\alpha = 0.05$), whereas no significant difference was found between the healthy and paresis groups ($p^*_{healthy,paresis} = 0.135$, $\alpha = 0.05$). These results indicate that temporal stability is significantly altered in polyp patients compared to healthy and paretic subjects, while no significant difference is observed between healthy and paretic individuals. Finally, Figure 6 (b.4) presents the mean lateral phase difference $\Delta \overline{\Theta }$ for the three clinical groups. Compared to the healthy subjects, the mean lateral phase differences appear slightly increased for both pathologic groups. However, the Kruskal-Wallis test did not indicate a statistically significant difference between the three groups ($p^* = 5.33 \cdot 10^{-2}$, $\alpha = 0.05$). As no significant group effect was found, no post-hoc tests were conducted. Thus, the presence of the investigated pathologies does not appear to affect the lateral phase synchronity of VF vibration in a characteristic way. To complement these findings, the corresponding PVG-based parameters were also analyzed for the clinical groups (Table 1). While the overall patterns of group differences align with those observed for the LVG-based parameters, differences in variability and central tendency can be observed. To further quantify the ability of both approaches to distinguish between clinical groups, effect size analyses were conducted using the absolute values of Cliff’s Delta. The results indicate that the LVG-based measures generally yield larger effect sizes compared to their PVG-based counterparts. The effect size for LVG was 0.44 ± 0.24 (0.41), whereas for PVG it was 0.33 ± 0.27 (0.26), corresponding to an approximately 58% greater median effect for LVG. Additionally, the interquartile range of effect sizes was smaller for LVG, suggesting a more consistent differentiation between groups. Thus, LVG-based measures tend to capture group differences more distinctly than PVG-based measures.

**Table 1:** Detailed group-averaged results for the investigated measures from Figure 6 stated as: mean ± standard deviation (median).

		healthy females	healthy males	healthy	paresis	polyp
LVG	$\overline{RND}_{r}$ [%]	$6.70 \pm 1.73 (6.61)$	$6.94 \pm 2.63 (6.57)$	$6.79 \pm 2.07 (6.57)$	$5.01 \pm 2.63 (4.77)$	$2.13 \pm 1.20 (1.91)$
	$\overline{RND}_{l}$ [%]	$6.23 \pm 1.52 (6.24)$	$6.17 \pm 1.96 (6.14)$	$6.20 \pm 1.67 (6.15)$	$4.92 \pm 2.49 (5.61)$	$2.36 \pm 1.45 (1.79)$
	$shim_{r,l}$ [%]	$3.63 \pm 1.16 (3.47)$	$4.02 \pm 1.51 (4.24)$	$3.78 \pm 1.30 (3.62)$	$7.98 \pm 7.32 (4.42)$	$12.77 \pm 9.61 (11.05)$
	$\overline{Q}_{RND}$ []	$0.89 \pm 0.05 (0.90)$	$0.82 \pm 0.07 (0.80)$	$0.86 \pm 0.07 (0.87)$	$0.60 \pm 0.26 (0.72)$	$0.73 \pm 0.14 (0.69)$
	$jit_{r,l}$ [%]	$4.95 \pm 2.08 (4.76)$	$4.80 \pm 2.55 (4.44)$	$4.90 \pm 2.25 (4.65)$	$11.13 \pm 13.54 (5.21)$	$27.50 \pm 26.87 (18.05)$
	$\Delta \overline{\Theta }_{r,l}$ [rad]	$0.50 \pm 0.42 (0.32)$	$0.40 \pm 0.29 (0.31)$	$0.46 \pm 0.38 (0.32)$	$0.80 \pm 0.56 (0.71)$	$1.07 \pm 1.04 (0.65)$
PVG	$\overline{RND}_{r}$ [%]	$8.72 \pm 2.20 (8.60)$	$8.66 \pm 3.38 (8.20)$	$8.70 \pm 2.65 (8.60)$	$6.96 \pm 3.08 (7.07)$	$3.46 \pm 1.56 (3.57)$
	$\overline{RND}_{l}$ [%]	$9.54 \pm 3.04 (9.33)$	$9.13 \pm 2.99 (8.05)$	$9.39 \pm 2.98 (8.96)$	$6.99 \pm 3.44 (8.53)$	$4.54 \pm 4.05 (3.76)$
	$shim_{r,l}$ [%]	$6.52 \pm 2.93 (5.42)$	$6.56 \pm 3.33 (7.10)$	$6.54 \pm 3.06 (5.79)$	$12.78 \pm 9.10 (9.85)$	$15.56 \pm 14.67 (12.36)$
	$\overline{Q}_{RND}$ []	$0.86 \pm 0.08 (0.88)$	$0.80 \pm 0.10 (0.82)$	$0.84 \pm 0.09 (0.87)$	$0.61 \pm 0.24 (0.71)$	$0.58 \pm 0.21 (0.60)$
	$jit_{r,l}$ [%]	$6.68 \pm 4.48 (5.51)$	$6.52 \pm 5.62 (4.72)$	$6.62 \pm 4.89 (5.43)$	$16.80 \pm 19.43 (6.95)$	$21.49 \pm 25.29 (8.44)$
	$\Delta \overline{\Theta }_{r,l}$ [rad]	$0.53 \pm 0.42 (0.39)$	$0.38 \pm 0.27 (0.33)$	$0.47 \pm 0.38 (0.38)$	$0.61 \pm 0.55 (0.48)$	$0.72 \pm 0.83 (0.43)$

### LVG analysis of voice onset

Non-stationary phonations are particularly valuable not only for diagnosing functional voice disorders but also for research, as they provide deeper insights into voice production. Thus, this analysis focused on exploring the potential of LVG-based analysis for reliably representing complex non-stationary phonation paradigms. For this purpose, a healthy subject (m, 30 yrs) was asked to perform two different voice onsets: a ’normal’ and a ’hard’ one. The HSV sequences each comprised a total of 1,400 frames. Quantitative analysis focused on the three phases of the voice onsets: the pre-phonatory phase, the onset phase, and the sustained phonation. These phases were identified based on the GAW. As the first phase, the pre-phonatory phase with VF adduction comprised in this work a fixed interval of the 1,000 consecutive frames (250 ms) before the onset of the first VF oscillations. The second phase, the onset phase, covered the interval between the onset of the first VF oscillations and the first oscillation cycle with full glottal closure. Finally, the third phase, the sustained phonation included the remaining part of the HSV sequence with all oscillation cycles of the sustained phonation.

**Fig. 7:**
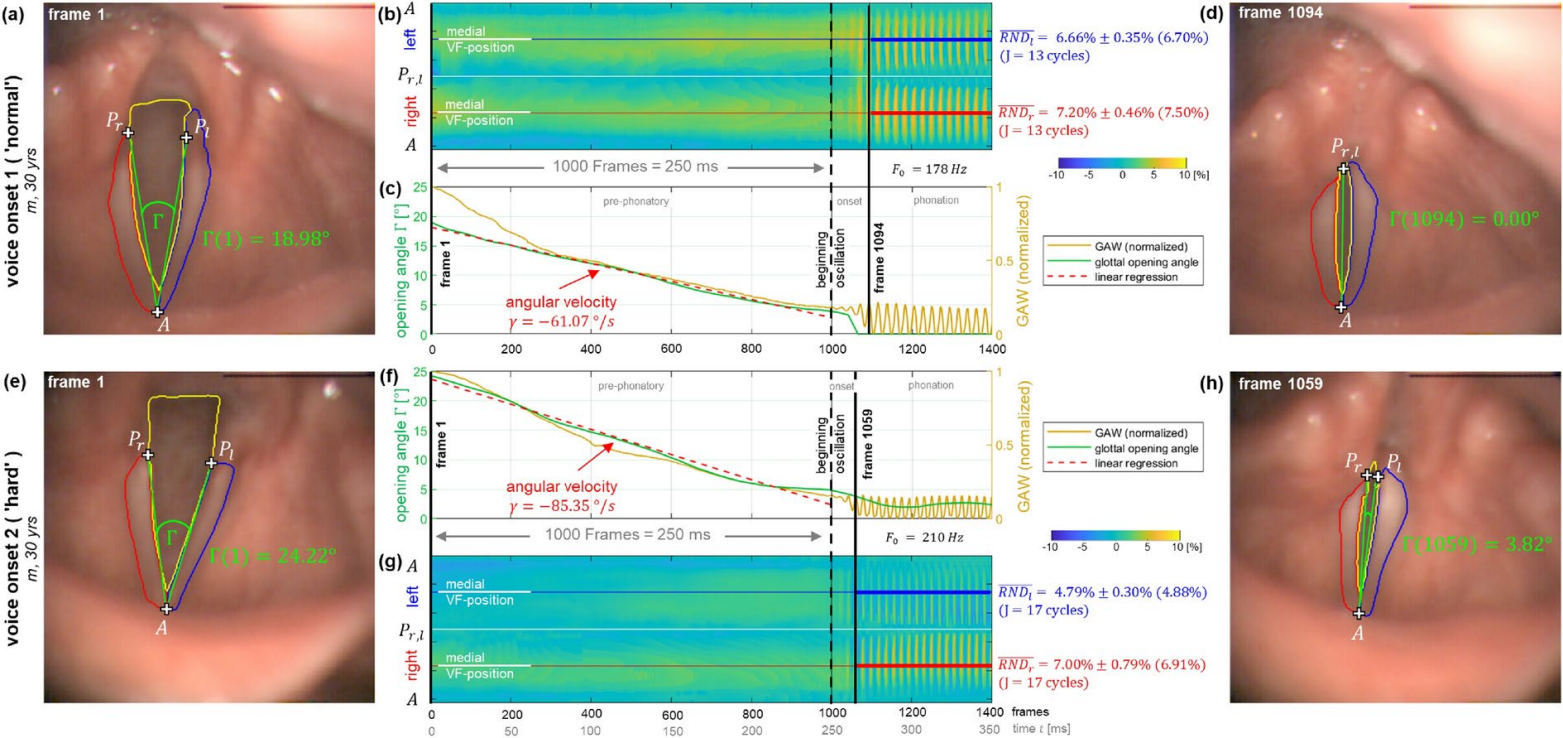
Two voice onsets performed by the same healthy subject (m, 30 yrs). Voice onset 1 shows a ’normal’ voice onset at a comfortable pitch and loudness (**a**-**d**). For the second recording, the subject was asked to perform a ’hard’ voice onset (**e**-**h**). Both sequences comprise a total of 1,400 frames, showing the pre-phonatory phase with VF adduction, the onset phase with the first VF oscillations, and sustained phonation. For each sequence, VF adduction during the 1,000 consecutive frames (250*ms*) prior to the onset of the first VF oscillations was analyzed using linear regression over the glottal opening angle in time $\Gamma (t)$. Frames from the pre-phonatory situation (a,e) and the phonatory situation at maximum glottal opening (**d**,**h**) are depicted, where $\Gamma (t)$ indicates the VF opening angle enclosed by both vibrational axes. The corresponding LVGs (**b**,**g**) and the visualization of the VF opening angle combined with the GAW (**c**,**f**) provide insights into the transitions during VF adduction. The ’hard’ voice onset exhibits a higher angular velocity of the VF opening angle $\gamma$, while both sequences show comparable relative normalized deflections ($RND_{r,l}$) during phonation.

Figure 7 illustrates both voice onset sequences. Panels (a,e) show the first frame of each captured sequence, representing the pre-phonatory situation, and panels (d,h) show a frame from the maximum glottal opening state during sustained phonation. The points $P_{r,l}(t)$ and *A*(*t*) were determined based on the segmentation results and defined the VF individual vibrational axes. The change of the VF opening angle $\Gamma (t)$, which is enclosed by both vibrational axes, was analyzed over time (Fig. 7 c,f). The velocity of VF adduction was quantified using the average angular velocity $\gamma$, which was determined by linear regression of $\Gamma (t)$ over a 250*ms* time interval during the pre-phonatory phase that precedes the voice onset (start of oscillation) immediately. Futher, the normalized GAW is displayed as a reference, providing an impression of the overall vibratory behavior. The LVGs (Fig. 7 b,g) were used to analyze the RNDs at the medial VF position during sustained phonation.

The ’normal’ voice onset (voice onset 1) starts with a VF opening angle of $\Gamma (1) = 18.98^{\circ }$. During the 1,000-frame pre-phonatory phase with VF adduction, the VF opening angle decreases at an average angular velocity of $\gamma = -61.07{^{\circ }}/s$ toward the phonatory situation. Sustained phonation with a fundamental frequency of $F_0 = 178~\text {Hz}$ is achieved 94 frames (23.5 ms) after the onset of the first oscillatory VF movements. During sustained phonation, which comprised of $J = 13$ full oscillation cycles, both VFs show regular, symmetric and synchronous VF oscillations with $\overline{RND}_{l}(p=50\%) = 6.66 \% \pm 0.35 \% (6.70 \%)$, and $\overline{RND}_{r}(p=50\%) = 7.20 \% \pm 0.46 \% (7.50 \%)$, respectively, at a VF opening angle of $\Gamma (1094) = 0{^{\circ }}$ (mean ± standard deviation (median)).

At the beginning of the ’hard’ voice onset (voice onset 2), the VF opening angle is $\Gamma (1) = 24.22{^{\circ }}$ and decreases during VF adduction at an average angular velocity of $\gamma = -85.35{^{\circ }}/s$. With only 59 frames (14.75 ms) between the initial VF oscillations and reaching the stationary state of sustained phonation, the transient process is completed faster than in the ’normal’ voice onset. The sustained phonation phase comprised $J = 17$ full oscillation cycles, with a fundamental frequency of $F_0 = 210~\text {Hz}$. During phonation, both VFs oscillate regularly, symmetrically and synchronously, with RNDs of $\overline{RND}_{l}= 4.79 \% \pm 0.30 \% (4.88 \%)$ and $\overline{RND}_{r} = 7.00 \% \pm 0.79 \% (6.91 \%)$, respectively, at a VF opening angle of $\Gamma (1059) = 3.82{^{\circ }}$. The sustained phonations of both voice onsets exhibited RNDs within the range of normal VF vibration (see section (c) Cross-sectional comparison of LVG analysis between clinical groups).

## Discussion

Clinical examination of voice disorders usually includes endoscopic imaging of the VFs. As a state-of-the-art technique, laryngeal HSV-endoscopy is used to evaluate the regularity, symmetry, and synchrony of VF vibrations in real-time^9^. The high temporal resolution of HSVs generates large amounts of data. Consequently, unlike laryngeal video-stroboscopy, manual replay for evaluation of the HSV is not feasible in everyday clinical routine. Therefore, extracting relevant information from HSV recordings and transforming them into a compact and clinically meaningful representation is crucial to efficiently interpret the vast amounts of data and make it usable in everyday clinical routine.

A well-known representation is the so-called PVG, which has been shown to provide a compact and clinically meaningful representation of the VFs’ vibrational behavior as captured in HSV recordings^57^. For the construction of the PVG, the vibrational behavior of the VFs is derived based on the movement of the edges of the glottal opening, as captured in the HSV recordings. As a result, the PVG is restricted to representing the VFs’ vibrational behavior only within the region of the glottal opening. Two major limitations result from this restriction to the glottal opening: (a) ’Unknown range and location of glottal opening’ (Limitation 1) - the PVG can represent the VFs’ kinematics only within the range of the glottal opening and cannot provide information on its range or location, and (b) ’No consideration of the individual vibrational axes’ (Limitation 2) - the vibrational behavior can only be represented with respect to the glottal symmetry axis, which may not coincide with the VFs’ actual vibrational axes, potentially altering the morphology of the PVG’s motion trajectories. These limitations of the PVG impact the interpretation of vibrational behavior, particularly in terms of comparing different HSV recordings and applying the PVG to non-stationary phonation paradigms: Quantitative inter- and intra-individual comparability is hindered, as the VF deflections cannot be normalized properly due to the unknown length of the VFs. Additionally, non-stationary phonations cause considerable changes in the overall laryngeal configuration that affect the position and size of laryngeal structures. These changes, which impact the extent, position, and vibrational behavior of the VFs, cannot be fully captured based on the glottal opening alone.

In this work, we introduced a novel approach called ’Laryngovibrography’. The resulting LVG aims to overcome the limitations of the PVG by, for the first time, providing a normalized representation of the VFs’ vibrational behavior along their entire lengths. Similar to the PVG, the LVG leads to a single image representation that contains the VF dynamics from an entire HSV sequence. Geometric patterns within the LVG uniquely represent the underlying VF dynamics and are quite similar to those within the PVG. Therefore, clinicians familiar with interpreting PVGs will also easily and intuitively understand the LVG representation. Previously, the geometric PVG patterns were shown to be in concordance with the ELS classification scheme of glottal closing patterns^25,47^. Since the LVG is essentially an extension of the PVG onto full length of the VFs, this also applies to the geometric LVG patterns.

This work focused on four main aspects of the introduced LVG representation. First, the overall differences between the PVG and LVG representations of the VFs’ vibrational behavior were investigated to assess whether the LVG complements the PVG. Following that, three aspects of the clinical application of the LVG were explored in frequent analysis scenarios, focusing on both intra- and inter-individual comparisons of HSV recordings, as well as evaluating the applicability of the LVG to non-stationary phonations.

### Comparing PVG and LVG

Due to its restriction to the region of glottal opening, the PVG cannot provide any information on the range or the location of the glottal opening. Furthermore, the vibrational behavior can only be represented with respect to one reference axis, the glottal symmetry axis. By extending the analysis of the VFs’ vibrational behavior to their entire length, the LVG overcomes these two limitations of the PVG resulting from its restriction to the glottal area. Since the LVG is based on the segmented VFs’ tissues itself, it provides direct information about the VFs’ vibrational behavior, rather than focusing solely on the glottal opening between them.

The LVG also reliably maps regions with permanent VF closure, regardless of whether these regions result from the individual’s physiology or any pathology. This ensures that the same positions along the VFs are always mapped to the same motion trajectories in the LVG. Additionally, since the LVG representation can provide information on both the location and relative extent of the glottal opening, it improves comparability between different recordings.

This was demonstrated in a sequence from a subject diagnosed with a unilateral VF polyp, where it was shown that organic lesions may be missing in the PVG, while they are included in the LVG. This case further demonstrates, that the LVG can be used to quantify the location and extent of organic lesions. Hence, the LVG facilitates follow-up examinations and the evaluation of treatment outcomes, which was previously in the PVG only possible to a limited extent. As a result, the LVG can be seen as a valuable extension of the PVG.

While the PVG is still one of the most comprehensive representations of VF dynamics, Kist et al. recently proposed an ANN-based approach to improve the representation of vibrational behavior further by more accurately retrieving the glottal symmetry axis from the segmented glottal area^104^. And while this data-driven alternative to traditional methods improves the representation of the vibrational behavior in the PVG, cases without dorsal contact of the VFs indicate that measuring VF deflections not perpendicular to their vibrational axes can alter the morphology of the resulting motion trajectories. Thanks to the additional segmentation of the VFs’ tissues, it is now possible to define individual vibrational axes for each VF, improving the representation of the vibrational behavior. By integrating this information on the VFs’ tissues into the analysis, the LVG represents a paradigm shift toward analyzing the VFs’ actual vibrational behavior, moving beyond just the opening-closing pattern of the glottis. This is particularly important in recordings from subjects with incomplete glottal closure, as demonstrated in the case of a subject with a VF polyp.

While this study primarily focuses on introducing the LVG representation, a systematic quantitative comparison of its diagnostic value over the PVG representation, including measures such as ROC analyses, will be the subject of future research.

### LVG analysis of repeated measures within a healthy subject

The PVG captures the distances between the VF edge and the glottal symmetry axis. However, these distances can vary considerably during different examinations depending on the anatomical conditions and zoom settings of the HS camera used. As a result, the VF deflections registered by the PVG are only comparable to a limited extent, as normalization is only possible to the glottal length.

By introducing VF deflections that are normalized to the length of the VFs’ tissues, the LVG aims to improve quantitative intra- and inter-individual comparisons of VF deflections. Thus, the potential of an LVG-based analysis for intra-individual comparisons was evaluated using repeated HSV recordings of sustained phonations from the same healthy subject. High conformity between the computed LVGs demonstrated that the presented approach comprehensively captures the vibrational behavior along the entire length of the VFs. The recordings from repeated sustained phonation showed only minor variations in the overall vibrational behavior. As the investigated healthy subject was not a trained singer, these variations likely resulted from slight changes in pitch, loudness, or the endoscope position inside the oropharynx.

The investigated motion trajectories showed high conformity, with their RNDs being approximately in the same range. However, this work focused solely on the normalized VF deflections at the medial VF position. In contrast, a previous study by Lohscheller et al.^27^ examined the location of the maximum RVA across the anterior-posterior extent of the glottal opening. In a cohort of 30 healthy women and 30 healthy men, they found that the maximum VF deflection occurred at 41% and 46%, respectively, along this extent. Given that reliable and automatic extraction of VF’ tissues from HSV recordings has only recently become possible^85^, there are currently no data available on the location of the maximum VF deflection concerning the full extent of the VF’ tissues. Consequently, the RND values in this work are only partially comparable to those in Lohscheller et al., as this work analyzed them for the first time along the entire lenght of the VFs.

Moreover, it was shown that in some cases, the RND might be negatively affected by overshooting behavior during the closed phase. This phenomenon occurs when both VFs oscillate together beyond their vibrational axes toward one side, which affects the computation of $RND_{r,l}$. It was demonstrated that this overshooting behavior also affects the amplitude stability, as the shimmer increased for the VF toward which both VFs oscillated together. In contrast, the temporal stability did not appear to be notably affected, with the slight variations in jitter likely attributable to the subject not being a trained singer. Besides the VF amplitudes themselves, asymmetries between the left and right VFs are of particular interest for the clinical interpretation of VF vibration. Despite the overshooting behavior, $\overline{Q}_{RND}$ indicated a high degree of lateral amplitude symmetry for all three sequences. Furthermore, lateral phase asymmetries in VF vibration were assessed using lateral phase differences. Various reasons for these lateral phase asymmetries have been discussed^13,35,105–107^, and it has even been demonstrated that these asymmetries, to a certain extend, are still common in healthy speakers^13,35,105^. However, only one study has performed a quantitative evaluation on lateral phase asymmetries based on the PVG. As part of a study on early-stage discrimination of malignant and precancerous VF lesions, Unger et al. investigated lateral phase differences computed from the PVG, specifically within the range of the glottal opening, and demonstrated that this parameter is useful for automated classification of voice disorders^35^. Analogous to the computation from the PVG, lateral phase differences can also be computed from the LVG, but with the advantage of evaluating the entire extent of the VF tissues. It was shown that the lateral phase differences observed in the repeated recordings within the range of the glottal opening were were consistent with those found in healthy controls in the study by Unger et al.^35^. However, when the entire VF length, including regions with permanent VF contact, was considered, the lateral phase differences increased considerably. This might be due to the minimal lateral displacement in these regions, where smaller or absent vibratory movements may introduce more variability or noise in the phase relationship. However, further investigation is needed to fully understand the underlying cause of this effect.

The overall high agreement of the VFs’ vibrational behavior observed in this intra-individual comparison of LVGs suggests that the approach can reliably represent inter-individual comparisons as well. Given that normative data represent an essential basis for a LVG-based evaluation of the VFs’ vibrational behavior, future studies should focus on collecting reference data from both healthy females and males. Additionally, investigating the intra-variability of normalized VF deflections in a larger group of healthy subjects is necessary to establish confidence intervals for RNDs in both intra- and inter-individual comparisons, and to validate the presented approach clinically.

### Cross-sectional comparison of LVG analysis between clinical groups

Since the previous analysis on repeated recordings demonstrated the reliability of the LVG approach, this analysis assessed its potential for inter-individual comparisons by analyzing the LVG representations of HSV recordings from three clinical groups. The quantitative evaluation and interpretation of LVGs require reference data from clinical groups. While previous studies intensively evaluated the spatiotemporal vibrational behavior of VFs using PVG-based parameters^27,48,98,108^, corresponding reference data for LVG-based parameters have yet to be established. To address this, a dataset comprising 66 clinical HSV recordings was analyzed to provide an initial understanding of the inter-individual variation in LVG-based measures for both normal and pathological VF vibrations. The performed analyses focused on various aspects of the VFs’ vibrational behavior. The vibrational behavior was investigated at the medial VF positions with respect to magnitude of VF deflection, amplitude stability and lateral VF vibration amplitude symmetry, as well as temporal stability of VF vibration and lateral phase synchronity.

For the healthy subjects, the RNDs clustered along the bisectrix, ranging from about 3% to 12%, with no statistically significant differences between the sexes, although slightly more RND variation was observed in healthy males. In contrast to the healthy subjects, both the pareses and the polyps exhibited considerably different RNDs. The large variability between the paretic subjects is likely related to the fact that, in this work, no further distinction was made regarding the muscular tonus or the posture of the affected VF. The increased variability observed in LVG-based measures for subjects with VF paresis (Fig. 6) reflects the underlying physiological condition rather than segmentation inaccuracies. Specifically, unilateral VF paresis with reduced muscular tonus leads to greater variability in vibrational behavior, which is successfully captured by the LVG. And since only the RND at the medial VF position was investigated here, it should also be noted that, for the polyps, the RND might, in some cases, be influenced by the location and extent of the lesion. However, for the pathologies, no relationship could be identified between the RND and the affected VF, as stated in the patient records.

The observed variation in the location of the medial PVG trajectory across clinical groups highlights a key methodological difference between the PVG- and LVG-based approaches. Due to the PVG’s restriction to the glottal opening range, PVG-based measures depend on individual anatomical conditions and may shift with pathology. In contrast, the LVG provides a fixed spatial reference, defined by VF length, ensuring a more standardized evaluation of vibrational behavior. These methodological differences are also reflected in the observed variance of PVG- and LVG-based parameters. Levene tests revealed significantly higher variance for PVG-based shimmer, which is likely due to the varying evaluation position along the glottal opening range. Interestingly, a similar increase in variance was found for PVG-based jitter, suggesting that temporal stability measures may also be indirectly influenced by positional variability. In contrast, no significant variance differences were observed for lateral amplitude symmetry or phase synchrony, indicating that these parameters are less affected by the choice of evaluation position. These findings highlight that while PVG-based measures are more sensitive to individual anatomical differences, LVG-based measures provide a more standardized assessment of vibrational stability.

Amplitude stability throughout the HSV sequence was confirmed for all healthy subjects, with no statistically significant differences related to sex. This finding is in concordance with the results of Inwald et al., who did not find any statistically significant differences in shimmer between the sexes, neither for the GAW-based shimmer nor for the motion trajectories at medial PVG position^98^. For the clinical groups investigated here, amplitude stability was shown to be affected by the presence of any of the conditions, which is in agreement with other studies on GAW-based shimmer^49,98^ as well as on PVG-based shimmer^98^. While an additional mass on the VFs had a strong impact on amplitude stability, the results also indicate that paresis significantly affects this parameter, albeit in a different manner. This aligns with previous studies reporting increased variability in vibrational behavior in paretic subjects, which has been associated with asymmetric vibrational patterns and irregular glottal closure dynamics^13,98,106^. However, it should be noted that in this study, both pathologies led to significant reductions in amplitude stability, but the underlying mechanisms might differ. These findings suggest that in the LVG-based representation, both paresis and an additional mass on the VFs strongly impact amplitude stability, albeit presumably through different mechanisms. While this observation aligns with previous studies using PVG- and GAW-based measures, the present study provides the first evidence of this effect in LVG-based analyses. Further investigations with a broader clinical dataset could help to better characterize these effects.

Moreover, it was shown that lateral amplitude symmetry is affected by the presence of paresis, while no statistically significant reduction was observed in the polyp group. This suggests that neuromuscular impairments have a stronger impact on vibrational asymmetry than structural lesions. However, the healthy subjects also demonstrated that lateral amplitude symmetry appears to be influenced by sex. A similar observation was made by Inwald et al. for lateral amplitude symmetry at the medial PVG position^98^. In the past, other research groups have also described asymmetries in VF vibration, even among healthy subjects, which also affect VF vibration amplitudes^13,35,105–107^. Thus, the slight variations in amplitude stability observed here, as well as the deviations from the bisectrix that lead to statistically significant differences in lateral amplitude symmetry between the groups, can likely be considered normal. This is further supported by the repeated recordings from a healthy male, which demonstrated that overshooting behavior leads to asymmetries in RND (see section (b) LVG analysis of repeated measures within a healthy subject).

Similar to amplitude stability, no significant sex-related differences in temporal stability were found for the healthy subjects. For the clinical groups, temporal stability was significantly altered in polyp patients compared to both healthy and paretic subjects, whereas no significant difference was found between healthy and paretic individuals. While Inwald et al. reported increased perturbation measures in patients with functional dysphonia and recurrent laryngeal nerve paralysis at the medial PVG position^98^, the present study found significant alterations only in patients with unilateral VF polyps. This difference may be attributed to the distinct underlying pathophysiological mechanisms of neuromuscular vs. structural pathologies. Moreover, Inwald et al. used a slightly different definition of perturbation measures, making direct numerical comparisons difficult.

The lateral phase differences observed for the healthy subjects were comparable to the healthy controls from the study by Unger et al.^35^ but showed some variability within the investigated groups. Although Unger et al. demonstrated that lateral phase difference is useful for the early-stage discrimination of malignant and precancerous VF lesions^35^, the present findings suggest that lateral phase synchronity remains largely unaffected by the pathologies investigated in this work.

The analyses offered an initial understanding of LVG-based measures across different clinical groups and highlighted the LVG’s potential to enable more reliable comparisons, which are essential for accurate quantitative evaluation in follow-up examinations. The results further suggest that the LVG representation of VFs’ vibrational behavior may be useful for the detection of pathologies. Effect size analyses reinforce this potential, showing that LVG-based measures differentiate between clinical groups more distinctly than PVG-based measures. The larger and more consistent effect sizes suggest that this approach captures clinically relevant differences with greater robustness, supporting its applicability for objective voice assessments. Future studies should explore this in greater depth, possibly by including a larger portion of the LVG (e.g., the medial third) rather than focusing solely on the medial VF position. While this study demonstrated the clinical applicability of LVG using rigid endoscopy at 4,000 fps, the approach itself is not inherently limited to this setup. Since PVG-based methods have been successfully applied across different framerates and both transorally and transnasally^11,16,109,110^, the LVG is expected to be similarly applicable. However, as with other analysis techniques, lower frame rates reduce the temporal resolution, which can affect the ability to capture fast oscillation dynamics within and between cycles^111^.

### LVG analysis of voice onset

The final analysis focused on the applicability of the LVG to non-stationary phonation paradigms. To explore the potential of an LVG-based analysis for non-stationary phonations, exemplarily, two voice onsets from a single healthy subject were investigated. Such comprehensive quantitative analyses of voice onset are of particular interest in clinical laryngology, as voice onset abnormalities, such as VF vibration onset delay, have been reported in patients with spasmodic dysphonia^112,113^, muscle tension dysphonia^114^, and Parkinson’s disease^115^.

To date, various studies have investigated voice onset^23,116–126^. However, assessment of the VF opening angle has so far been performed either manually^116,118^ or semi-automatically^123,126^. Due to its time-consuming nature, this is not feasible in clinical routine, which is why the degree of VF abduction or adduction is still often rated subjectively using ordinal scales^127,128^. Until now, studies of VF abduction and adduction processes during maneuvers such as voice onset, voice offset, or throat clearing have been limited by the time-consuming extraction of the VF opening angle. Recently, Adamian et al. presented a Deep Learning-based tool that aims to overcome this limitation by fully automatically tracking the VFs’ edges via landmark detection and estimating the VF opening angle from their localization^127^. However, their study used videolaryngoscopic data (29 fps) that were acquired transnasally with a flexible endoscope.

In the present work, two voice onsets on sustained phonation of the vowel /æ/ from a single healthy male subject were investigated: a ’normal’ and a ’hard’ one. The focus was to explore the potential of the proposed LVG for non-stationary phonations and to evaluate whether it is capable of simultaneous tracking the VF transition via the VF opening angle enclosed by the two VF individual vibrational axes. Unlike Adamian et al., we used a high-speed camera with a temporal resolution of 4,000 fps coupled to a rigid endoscope.

Until recently, automated detection of VF tissue in HSVs was not possible. Thus, in previous studies, the anterior commissure was either determined by the anterior end of the glottal opening^118,123^ or approximated as the intersection of linear fits of the VF edges^126,127^. Since the U-LSTM used here allows for the extraction of VFs tissue itself^85^, direct identification of the anterior end of the VFs is now possible. Therefore, in this work, the anterior end of the VFs was used as an approximation of the anterior commissure and as an anchor to quantify the VF opening angle. Consequently, the VF opening angles reported here are mostly not directly comparable with those from previous studies.

Comparison of the VF opening angle is further complicated by the use of different endoscope types, which may lead to variations in viewing angles on the VF vibration plane. Thus, the glottal opening angles found in this study are likely not directly comparable to the works by Dailey et al.^118^, Iwahashi et al.^123^, Cadiz-Diaz et al.^126^, and Adamian et al.^127^ who, unlike us, used flexible endoscopes. Additionally, Pietruszewska et al. argues that rigid HSV-endoscopy, unlike flexible HSV-endoscopy, induces a form of forced phonation, which alters the observed laryngeal configuration^100^. This effect might further complicate comparisons between different studies.

Large differences in maximum VF opening angles are also evident between these studies, ranging on average from 25$^{\circ }$ to 65$^{\circ }$. Direct comparison is further complicated by the fact that our HSV recordings comprised a fixed interval of 1,000 frames (250 ms) prior to the onset of VF vibrations. Based on Kymograms, Eysholdt et al. argued that the VF transition from the respiratory to the phonatory situation is an aperiodic process that lasts about 300-500 ms^117^, although this highly depends on the individual phonation onset. Hence, it is possible that the maximum VF opening angle measured in this study does not reflect maximum VF adduction.

In agreement with the findings of Dailey et al.^118^, we observed higher angular velocities during VF adduction for the hard voice onset compared to the normal voice onset, but with somewhat lower angular velocities than they reported. Furthermore, Iwahashi et al. reported that the change of the VF opening angle over time follows a sigmoid-shaped curve^123^. However, we did not observe such a sigmoid-like change in the VF opening angle, which may result from a limited interval prior to voice onset available in this study.

According to Woo et al., the voice onset phase itself lasts three to five oscillation cycles before the VFs reach a steady-state and sustained phonation^125^. The same observations were made for the voice onsets presented here. Both exhibited four oscillation cycles during onset prior to the first oscillation cycle with full glottal closure that initiates the phonatory phase.

During phonation, the retrieved RNDs for the ’normal’ and the ’hard’ voice onset were within the range of the healthy males from the previous analysis. The ’hard’ voice onset showed slightly reduced RNDs compared to the ’normal’ onset. As the investigated subject was not a trained singer, this is likely due to the slightly higher $F_0$ and the resulting altered laryngeal tensions.

This analysis of non-stationary phonation using the LVG-based approach demonstrated that the LVG extends prior work by enabling, for the first time, fully automated and simultaneous registration of the VFs’ vibrational behavior and the VF opening angle during VF adduction. The proposed approach was shown to be capable of processing large amounts of data from HSV while providing reliable results even for long, non-stationary sequences. These initial findings from an LVG-based analysis of non-stationary phonation suggest its potential to provide new insights into both physiological and pathological mechanisms of voice production. Given the clinical relevance of voice onset dynamics, particularly in neurological and neurogenic voice disorders such as Parkinson’s disease, future studies will focus on validating LVG-based assessments in these patient populations. By enabling a comprehensive analysis of transient phonation paradigms along the entire VF length, the LVG may facilitate the objective characterization of phonatory impairments, aiding diagnosis, monitoring of disease progression, and tracking of therapy outcome.

## Conclusion

Research on voice production and clinical diagnosis of voice disorders requires imaging of the laryngeal structures and the vibrational behavior of the VFs. Laryngeal HSV-endoscopy is a state-of-the-art technique for investigating physiological and pathological VF vibrations. However, the large amounts of data generated by HSV makes its evaluation time-consuming, which is particularly problematic for clinical routine.

In this study, we introduced a novel approach called Laryngovibrography, which extends the prior Phonovibrography approach by, for the first time, analyzing the vibrational behavior along the entire length of the VFs. The so-called Laryngovibrogram provides a compact single-image representation that contains the normalized VF dynamics from an entire HSV sequence. A key feature of the LVG is the normalization of VF deflections, allowing for consistent and comparable analysis across different recordings and subjects. Unlike PVG-based measures, which are tied to the glottal opening and thus influenced by individual anatomy and pathology, the LVG evaluates vibratory behavior relative to the full VF length. This provides a standardized spatial reference, reducing inter-individual variability. Effect size analyses indicate that LVG-based measures tend to differentiate differentiate between clinical groups more consistently than PVG-based measures, although further validation is required to confirm these findings. Thus, the Laryngovibrogram extends the traditional Phonovibrogram by offering a more detailed representation of VF regions that remain closed during phonation, as well as precise data on the size and location of the glottal opening. As such, the proposed approach represents a paradigm shift in voice research toward analyzing the actual vibrational behavior of the VFs’ entire length, rather than focusing solely on glottal opening dynamics. Furthermore, the LVG representation shows potential to objectify clinical diagnosis by providing a more detailed and holistic view of VF vibrational behavior. In particular, the LVG might complement existing diagnostic tools by facilitating quantitative assessments of vibratory impairments, thereby assisting clinicians in identifying and classifying pathological conditions. Future studies will need to explore whether the LVG can be used to document and track therapy outcomes, which is an essential prerequisite for evidence-based medicine. Additionally, the LVG could potentially be integrated into clinical workflows as an adjunctive tool for monitoring treatment progress, such as evaluating post-surgical recovery or the effects of voice therapy. The LVG also allows for simultaneous assessment of both the VF opening angle and the vibrational behavior of the VFs, which might be beneficial for studying voice onset irregularities in neurological disorders. By providing the basis for a comprehensive quantitative analysis of VF vibration and kinematics, the Laryngovibrography approach presented here holds great potential for the investigation of various phonation paradigms, which could lead to a better understanding of voice physiology and the characterization of pathologies. Moreover, improved intra- and inter-individual comparability of vibrational behavior enhances the reliability of clinical studies. By providing a normalized representation of the laryngeal dynamics along the entire lengths of the VFs, the LVG not only ensures consitency in analysis but may also promote the development of clinical support systems that utilize automated classification of voice disorders.

## Supplementary Information

Supplementary Information 1.

Supplementary Information 2.

## Data Availability

The datasets generated and analyzed during the current work are available from the corresponding author upon request. A subset of representative data used for generating the figures and validating the method has been deposited and is available via Zenodo (DOI: 10.5281/zenodo.15263300).

## Code availability

The custom code developed for the generation of the LVG and related analyses has been deposited and is publicly available via Zenodo (DOI: 10.5281/zenodo.15263300). The code includes all scripts required to reproduce the main results presented in this work. In addition, we provide a MATLAB toolbox that enables users to compute LVGs from segmentation results, allowing application to other HSV recordings.
