# Supplementary figures and images for: The Laryngovibrogram as a normalized spatiotemporal representation of vocal fold dynamics
Source: Sci Rep. 2025 May 12;15:16473. doi: 10.1038/s41598-025-00966-8 (PMC12069559; doi:10.1038/s41598-025-00966-8)

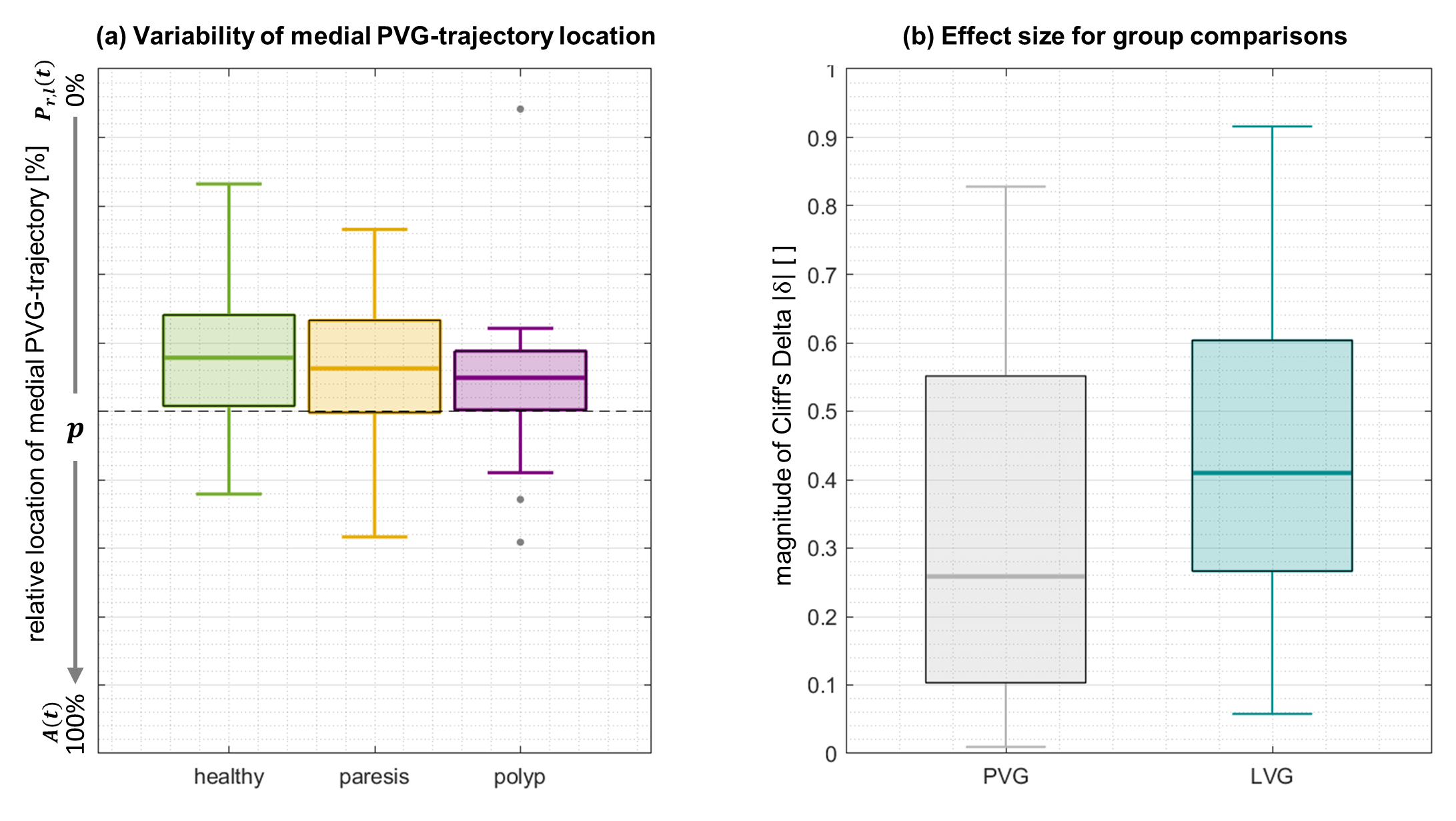

Supplement: Supplementary file 1 — Supplementary Information 1. [file 41598_2025_966_MOESM1_ESM.png]
