# Supplementary material for: The Laryngovibrogram as a normalized spatiotemporal representation of vocal fold dynamics
Source: Sci Rep. 2025 May 12;15:16473. doi: 10.1038/s41598-025-00966-8 (PMC12069559; doi:10.1038/s41598-025-00966-8)
Supplement: Supplementary file 2 — Supplementary Information 2. [file 41598_2025_966_MOESM2_ESM.docx]

Fig S1: Comparison of PVG- and LVG-based measures. (a) Variability of relative location of the medial PVG trajectory assessed relative to the VF length, as used for LVG construction, across both VF and all three clinical groups. Data are shown separately for healthy subjects, patients with unilateral VF paresis, and patients with unilateral VF polyps. (b) Effect size for group comparisons across clinical groups. The absolute values of Cliff's Delta were computed from all investigated parameters and pooled separately for PVG and LVG, illustrating the effect sizes for distinguishing between clinical groups.
